# Highly Controlled Parylene C Coating on Titanium for Invasive Biomedical Applications

**DOI:** 10.3390/mi17080953

**Published:** 2026-08-12

**Authors:** Sarra Riahi, Salim Braiek, Nathan Martins, David Bouville, Xavier Lafosse, Frédéric Mahut, Alain Bosseboeuf, Muriel Thomasset, Christophe David, Gwenael Becan, Bertrand Boutaud, Elie Lefeuvre, Mehdi Ammar

**Affiliations:** 1 Center for Nanosciences and Nanotechnologies (C2N), CNRS UMR 9001, University of Paris-Saclay, 91120 Palaiseau, Francealain.bosseboeuf@universite-paris-saclay.fr (A.B.); muriel.thomasset@cnrs.fr (M.T.);; 2 MISTIC SAS, Issy-les-Moulineaux, 92130 Paris, France

**Keywords:** parylene C, titanium implants, biomedical coatings, active medical devices, interfacial adhesion, accelerated aging, micro-encapsulation, corrosion protection

## Abstract

The rapid development of implantable medical electronics requires robust biocompatible coatings capable of ensuring long-term stability in aggressive physiological environments. Although Grade 1 titanium is widely used for its excellent mechanical properties and corrosion resistance, active implants require defect-free insulating coatings to prevent electrical leakage and metal ion release. This study presents a systematic evaluation of Parylene C (P-C) thin films deposited by the Gorham chemical vapor deposition (CVD) process onto implant-grade titanium substrates. Four coating thicknesses (1, 5, 10, and 20 µm) were deposited and characterized using complementary chemical, morphological, optical, and mechanical techniques. Contact-angle measurements confirmed uniform hydrophobicity (90.56 ± 1.86°), while FTIR and EDX verified the characteristic chemical composition of P-C. Reflectometry, ellipsometry, and interferometry demonstrated excellent thickness control and deposition reproducibility. Pull-off testing showed high initial mechanical integrity, with detachment forces ranging from 52 to 73 N. However, accelerated PBS ageing (21 days at 90 °C) induced significant degradation, particularly for thicker coatings, reducing pull-off forces to 19–42 N. Likewise, thermal-shock cycling (−80 °C to +220 °C) caused severe interfacial damage, decreasing the required detachment force to approximately 5.5 N for 20 µm coatings because of extensive cracking and delamination. These results demonstrate that Parylene C provides excellent conformal coverage and chemical stability on titanium but that its durability is significantly affected by prolonged hydrothermal ageing and extreme thermal loading. This study provides practical guidelines for the design of reliable encapsulation systems for active implantable medical devices and highlights the need for improved interfacial engineering through optimized adhesion-promoting layers or hybrid protective architectures.

## 1. Introduction

The rapid advancement of biomedical technologies has driven a growing demand for materials that can reliably function within the human body. Medical implantable devices, ranging from orthopedic implants and dental fixtures to neural probes and cardiovascular devices, must meet strict mechanical, chemical, and biological requirements [1]. These devices operate in complex biological environments and must maintain long-term performance without causing adverse reactions. Consequently, the selection and surface engineering of biomaterials are critical to ensure both functionality and biocompatibility.

In this context, Titanium (Ti) is extensively used in biomedical implants because it combines high tensile strength and low elastic modulus with excellent corrosion resistance and bone compatibility [2,3]. Its passive surface oxide (TiO_2_) provides outstanding chemical stability, enabling implantable titanium to integrate with bone without causing acute toxicity [4]. Plus, this same passivation gives Ti surfaces a fundamentally bio-inert character. Under harsh conditions the native oxide can locally break down, potentially releasing metal ions into tissue [5]. These factors motivate additional surface treatments to enhance implant performance. Therefore, polymeric coatings have been explored to further encapsulate titanium implants and improve their surface properties [6]. In practice, a wide variety of polymers have been used for device encapsulation such as Poly(methyl methacrylate) (PMMA), Cyclic olefin copolymer (COC), Chitosan, and Polydimethylsiloxane (PDMS), and parylene [6,7]. However certain properties can limit the performance of some of these polymers, for instance, PDMS, while biocompatible and easy to process, is porous and absorbs small molecules, which can compromise chemical stability and device reliability [8].

Within this group of protective polymers, parylene has gained significant attention as highly effective encapsulation material. Belonging to the poly-para-xylylene family, parylene is valued for its distinctive chemical vapor deposition (CVD) process, which yields thin, conformal, pinhole-free films with strong adhesion to diverse substrates [9]. Among the various parylene types [10], parylene C (P-C)-“poly(chloro-p-xylylene)” is by far the most widely used in biomedical applications [11]. Its single chlorine substitution gives a convenient balance of properties, and it has vastly improved moisture and gas barrier properties [12,13]. Therefore, it has been used as an encapsulant for neuroelectrodes [14], flexible retinal implants [15], as well as a dielectric in implantable capacitors and transistors [16].

Coating a titanium implant with parylene C promises a synergistic combination of properties. The titanium provides structural support and rigidity, while the thin polymer film provides an ultra-pure, inert interface to the body. Studies of parylene on metals have indeed reported dramatic reductions in corrosion currents when the polymer encapsulates the surface [17]. For example, recent reports highlight that parylene layers on stainless steel and titanium alloys improve longevity by preventing fluid ingress and ionic diffusion [18]. Such findings corroborate the fact that parylene C coating greatly enhances corrosion protection in contact with biological fluids [15,19]. The limitations described (ion release, corrosion, etc.) relate to alloys, whereas pure titanium does not have extensively all these limitations. Therefore, there is a real advantage to using parylene on active implants and on pure titanium as an electrical insulation layer, particularly to ensure that the titanium casing of the implant is fully or partially isolated from the electric field vectors potentially applied to the organs.

In this context, a fully systematic study of parylene C on bulk titanium for biomedical applications has not yet been reported. To the best of our knowledge, this work presents the comprehensive multi-technique evaluation of parylene C coatings on Grade 1 Titanium. We examine key properties: from the chemical composition and bonding at the interface, to the coating morphology and thickness, through mechanical characterization, to biomedical-oriented tests needed for invasive applications. Our results provide a comprehensive understanding of the parylene C/Ti interface, guiding future development of corrosion-resistant, bioinert coatings for critical medical implants.

## 2. Experimental: Materials and Methods

### 2.1. Sample Preparation

In this study, depositions were conducted on bulk titanium grade 1 substrates initially prepared as 4-inch wafers with a thickness of 300 µm. These wafers were subsequently diced into 10 × 10 mm square samples to facilitate uniform processing and characterization. Prior to deposition, the substrates underwent a standardized cleaning protocol designed to eliminate organic and particulate contaminants. This cleaning procedure involved ultrasonic baths in acetone and isopropanol, followed by thorough rinsing and drying under a nitrogen flow to ensure a residue-free surface.

Based on the deposited thicknesses, the samples were categorized into four separate batches: batch 1 [1 µm], batch 2 [5 µm], batch 3 [10 µm] and batch 4 [20 µm], allowing for comparative analysis of the impact of film thickness on the subsequent physicochemical and mechanical properties.

### 2.2. Parylene Deposition Process

The deposition of parylene is based on the Gorham process, a dedicated vacuum-based batch process that consists of three key steps: vaporization, pyrolysis, and polymerization onto the substrate at room temperature. Unlike conventional Chemical Vapor Deposition (CVD) processes, the Gorham process does not rely on high temperatures or reactive gases, allowing for the formation of uniform, pinhole-free coatings on a wide range of substrates, including temperature-sensitive materials.

The deposition of parylene films was conducted using the parylene Coater C30H ALD (SCS, Indianapolis, IN, USA and La Chaux-de-Fonds, Switzerland), a hybrid equipment integrating advanced multilayer coating technologies. This machine, originally provided by Comelec SA as Switzerland operations of SCS company (Indianapolis, IN, USA and La Chaux-de-Fonds, Switzerland), offers a unique combination of parylene and atomic layer deposition (ALD) capabilities, enabling precise and innovative coating processes.

Within the deposition chamber, the substrates are placed on a large rotating sample holder designed to ensure uniform exposure to the monomer flux, thereby promoting even parylene deposition across all surfaces.

Before the actual deposition process, two preparatory steps are typically performed in the same chamber to enhance film adhesion. First, the samples are exposed to an oxygen plasma treatment, which activates the surface. This is followed by the introduction of an adhesion promoter, the silane A-174, which forms a molecular bridge between the substrate and the polymer, significantly improving the adhesion of the deposited parylene layer.

Then the Gorham process begins with vaporizing the solid dimer at a temperature of 165 °C. These sublimated dimers then enter a pyrolysis oven heated to ~650 °C, where they undergo thermal cracking to produce highly reactive monomer vapors.

**Figure 1:**
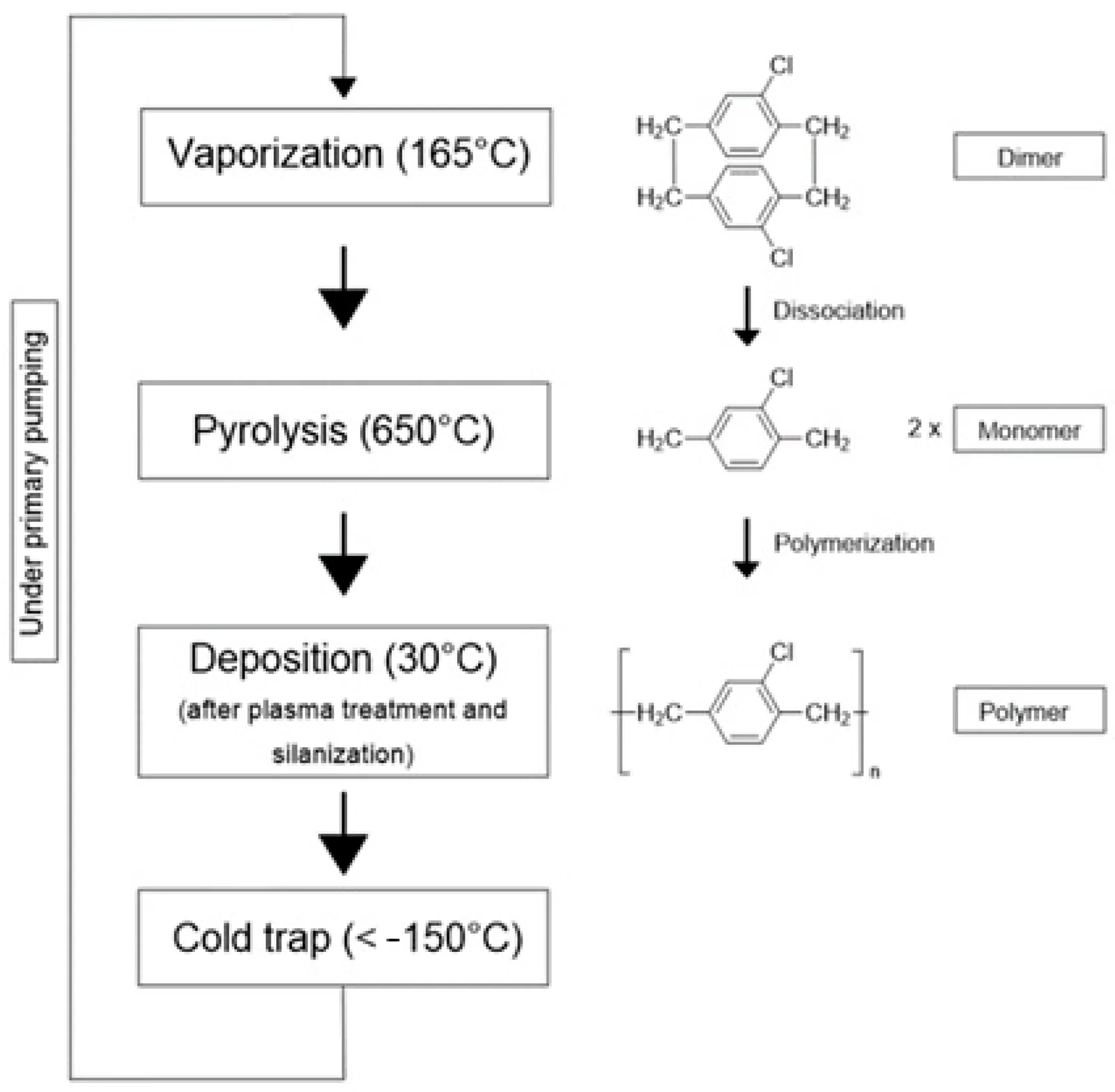
Schematic representation of a deposition process of parylene C with the respective chemical reactions.

The final step occurs in a room-temperature deposition chamber, where the gaseous monomers condense and spontaneously polymerize upon contact with any surface. This step elevates the pressure in the chamber from 3 µbar to over 16 µbar (with a maximum of ~20 µbar). Once the pressure exceeds this threshold, deposition starts, and it continues until the pressure drops back below 11 µbar, at which point the deposition stops (Figure 1). The process is completed by pumping out excess gases and removing them using a cold trap, typically employing liquid nitrogen (LN_2_).

This unique deposition method allows parylene coatings to be applied uniformly in a controlled environment, providing excellent encapsulation and conformal coverage on various surfaces and complex geometries.

The deposition process involved systematically varying the mass of the introduced dimer precursor to control the final film thickness. Multiple runs were performed under identical conditions, with the only variable being the amount of dimer introduced into the system. This approach enabled the fabrication of four parylene C thicknesses: 1 µm, 5 µm, 10 µm, and 20 µm, each deposited conformally on both sides of the samples.

### 2.3. Characterization of Parylene C Films Deposited on Titanium Wafers

The characterization was primarily conducted to evaluate the structural and functional quality of the deposited parylene C films. Multiple complementary analytical techniques were employed, and the resulting datasets were plotted using custom Python 3.9 scripts.

#### 2.3.1. Surface Wettability: Contact Angle Measurement

To evaluate the hydrophobicity of the deposited parylene C films. Surface wettability was assessed via static contact angle measurements using a Dataphysics OCA20 goniometer (DataPhysics Instruments GmbH, Filderstadt, Germany). A 1 µL drop of deionized water was dispensed at a rate of 0.5 µL/s, and each of 9 samples from batch 2 (5 µm thickness) underwent 5 to 10 individual measurements to ensure statistical reliability.

#### 2.3.2. Chemical Composition Characterization

To investigate the chemical composition of the parylene films, two complementary techniques were employed: Fourier Transform Infrared Spectroscopy (FTIR) and Scanning Electron Microscopy (SEM) coupled with Energy Dispersive X-ray Spectroscopy (EDX).

FTIR was utilized to identify the chemical bonds, and functional groups present in the parylene C films. Measurements were conducted on a sample from batch 2 in reflectance mode using a Varian FTIR spectrometer (Agilent, Inc., Les Ulis, France) equipped with a grazing angle accessory. This configuration allows non-contact analysis of the rigid titanium substrate while enhancing the interaction with the polymer surface. Spectra were acquired with a resolution of 8 cm^−1^ and processed using the Origin software 2023 providing adequate spectral detail to resolve narrow absorption features.

In addition, Surface elemental composition was assessed via a Magellan 400L/XHR Scanning Electron Microscope with integrated EDX system/FEI (Thermo Fisher Scientific, Inc., Landsmeer, The Netherlands). Operating at 10 kV and 0.2 nA, this configuration enables the detection of characteristic atomic peaks within the surface region of interest. The visibility of these peaks is governed by each element’s excitation energy and the system’s voltage and current thresholds, ensuring accurate qualitative and semi-quantitative elemental analysis. Atomic percentage data were obtained for a sample from batch 2 (5 µm thickness) which was subjected to 9 individual measurements.

#### 2.3.3. Thickness Measurements

To ensure the reliability of the thickness measurements, three techniques were employed: spectroscopic reflectometry, spectroscopic ellipsometry, and White-Light Scanning Interferometry (WLSI).

##### Spectroscopic Reflectometry

To perform thickness measurement analysis using reflectometry, a Filmetrics F20 Reflectometer (Filmetrics, Inc., San Diego, CA, USA) with a resolution of 0.3 nm was used. For this technique, 5 samples from each batch were measured, with 9 measurements taken on each sample.

##### Spectroscopic Ellipsometry

The Woollam M2000 Ellipsometer (J.A. Woollam Co., Inc., Lincoln, NE, USA) was used to determine the thickness of the parylene C films. The ellipsometer settings were adjusted, including the beam’s angle of incidence set at 70°, and subsequently identified the material under examination. Five samples from each batch were measured, with 3 measurements taken on each sample.

##### White-Light Scanning Interferometry (WLSI)

White light scanning interferometry WLSI is a non-contact optical measurement technique used for high-resolution three-dimensional surface profiling [20].

**Figure 2:**
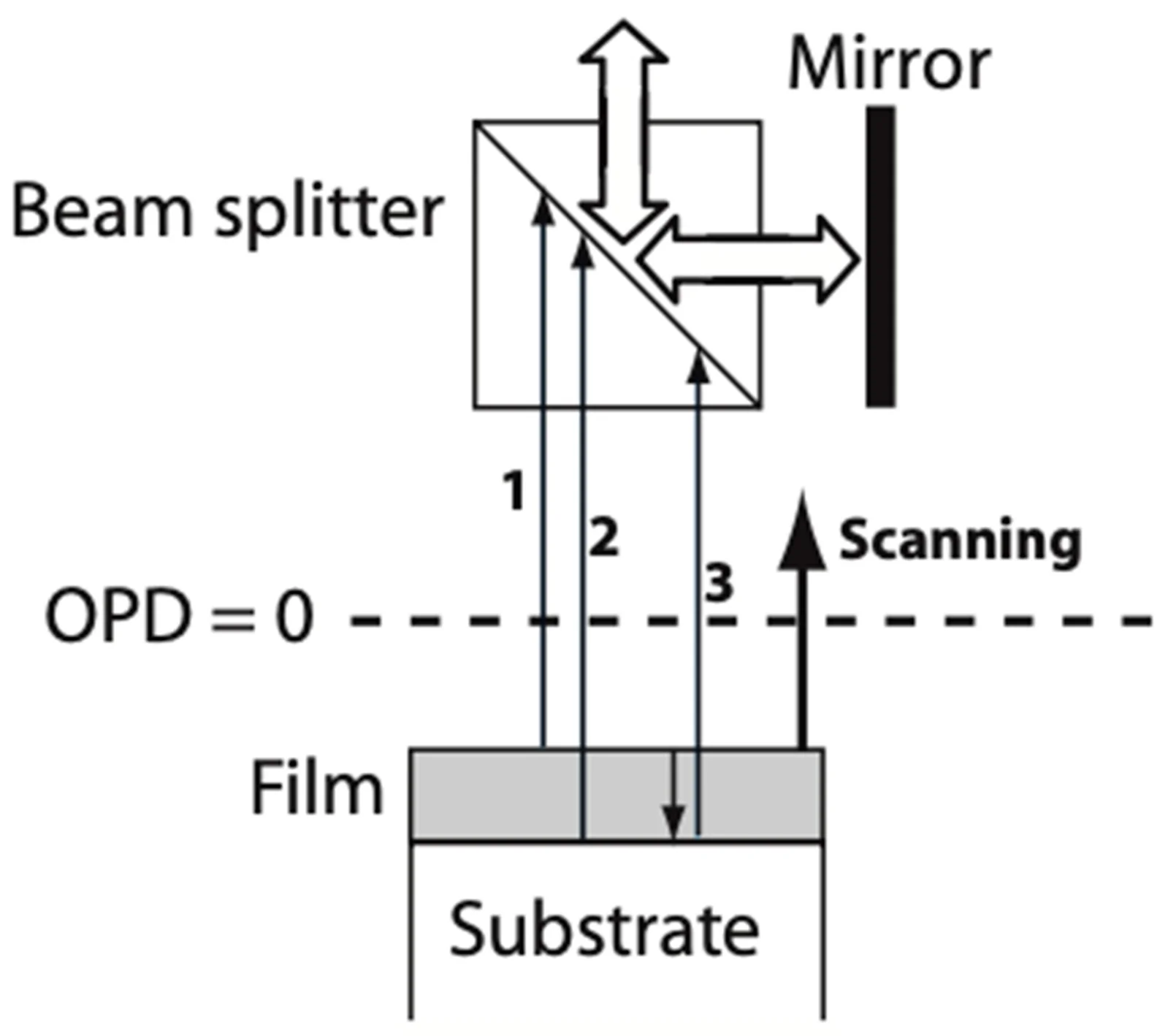
Principle of transparent film measurements by WLSI [22].

By employing WLSI, the intensity signal *I*, recorded by a camera pixel as a function of the altitude *z*, results from interferences between the reference beam and the light beams coming from the reflection on the top film surface, and from the reflection at the bottom interface (Figure 2). The interferometric signals were analyzed using custom MATLAB (MathWorks 13) code based on Fast Fourier Transform and peak detection algorithms. To measure the thickness of the parylene C films, a profilometer, MICROSURF 3D from Fogale Nanotech company (Fogale Sensors, Nîmes, France), was employed. For optical characterization, we used the refractive index value of 1.639 for parylene C [21]. For this technique, 5 samples from each batch were measured, with 9 measurements taken on each sample.

#### 2.3.4. Surface Morphology: Atomic Force Microscopy (AFM)

To gather insights into the surface morphology of parylene C films, Atomic Force Microscopy (AFM) was employed to achieve high-resolution surface imaging. For this analysis, the Park Systems AFM F40 (Park Systems Corp., Gwacheon, Republic of Korea) was used, operating in non-contact mode (NCM). Measurements were performed using a scan size of 10 × 10 µm^2^ at a scan rate of 0.5 Hz, providing a comprehensive understanding of the surface morphology across samples from batch 2.

### 2.4. Application Based Characterization: Tests for Implantable Devices

#### 2.4.1. Pull-Off Adhesion Test

**Figure 3:**
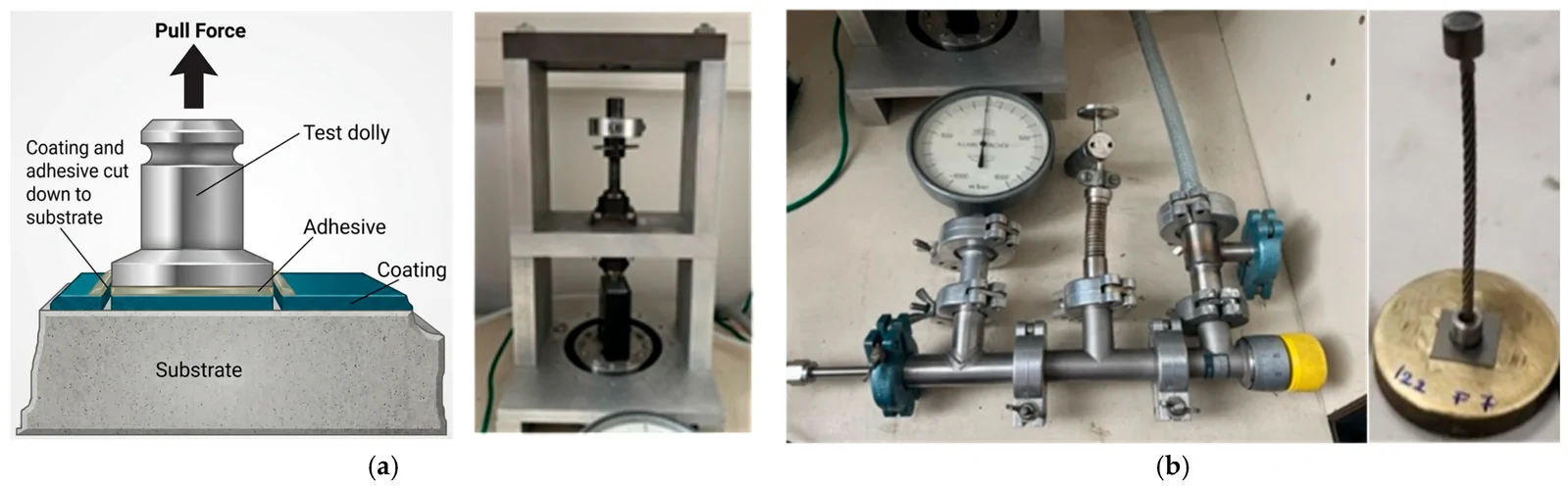
(**a**) Pull-off test principle [23]; (**b**) Home-built Pull-off test set up.

Pull-off test is an effective method for evaluating the comparative mechanical integrity of parylene C coatings to the substrate, titanium substrates in the case of this study. This technique (Figure 3a) applies tensile stress to the coating to measure its bond strength to the substrate [23].

The pull-off force was measured using a home-built (Figure 3b) adhesion setup that consists of a sample-fixing system glued to a brass roller on one end and a rod on the other. The rod is secured in a clamp. Force is applied using a pneumatic system that exerts a pulling force on the rod. A force sensor is employed to monitor the applied force over time. All the chips were glued onto brass rollers using the COLTOU [24] glue to be tested under force, and a pulling rod was glued onto the outer surface of the parylene film.

#### 2.4.2. Corrosion Test

Immersion test or accelerated PBS ageing test is a long-term experiment designed to simulate how components react when in contact with blood, which is viewed as a corrosive saline medium.

The components are immersed in a solution known as PBS (Phosphate-Buffered Saline). The salinity of this solution is carefully adjusted to prevent harm to biological elements, and it is tailored to achieve a salinity level of approximately 15 mS/cm, similar to the conductivity of blood [25].

Heating the solution accelerates its corrosive properties. For instance, a 21-day exposure at 90 °C is equivalent to an implantation period of 10 years at 37 °C [26,27]. Accelerated ageing was selected according to the Arrhenius approach commonly adopted for medical devices. The acceleration factor depends on the activation energy assumed for the dominant degradation mechanism. Consequently, the equivalence between 21 days at 90 °C and long-term exposure at physiological temperature should be regarded as an engineering approximation rather than an exact prediction. Furthermore, elevated-temperature exposure may activate hydrothermal degradation pathways that differ from those occurring under physiological conditions [26].

For this test, 5 samples of each parylene C batch were tested. Each sample was individually immersed in a 45 mL centrifuge tube containing PBS with a conductivity of 15.1 mS/cm. Four samples of each thickness were placed in a MEMMERT UN30 oven (Memmert GmbH and Co. KG, Schwabach, Germany) at 90 °C, while the fifth sample of each batch served as a control and was kept at ambient temperature.

Daily observations were performed on the selected samples to monitor any morphological or visual changes in the parylene film throughout the experiment. Upon completion of the exposure protocol, each sample underwent optical microscopy to characterize surface morphology, followed by adhesion tests (Pull-off test) to quantify the effects of corrosion and thermal variations on the film’s comparative mechanical integrity and mechanical integrity.

#### 2.4.3. Cyclic Thermal Shock

A thermal shock test is used to heavily stress components or materials to simulate aging conditions and evaluate the durability of packaging constraints, such as soldering on biomedical devices [28]. Sudden temperature changes will reveal defects in materials or adhesion issues. If defects are not initially visible (assuming a localized defect), they are expected to propagate, resulting in the formation of a crack. The current method for testing implantable components under thermal cycling is MIL-STD-202 Method 107G [29].

**Figure 4:**
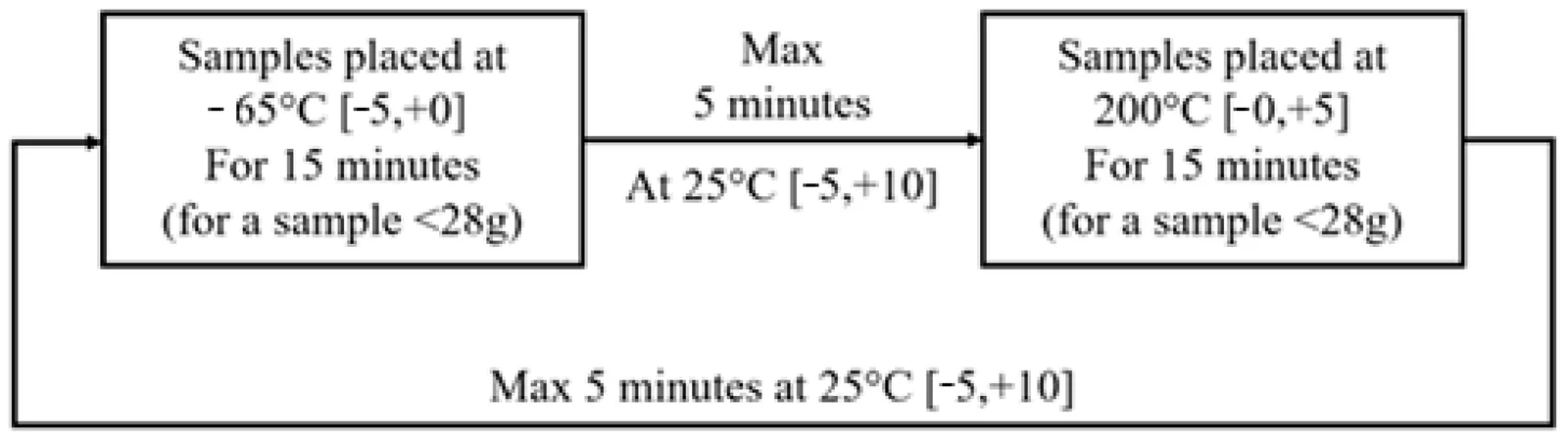
Thermal shock cycle.

The test involves 5 successive cycles composed as represented in Figure 4. For this study, 5 samples of each parylene C batch were exposed to temperatures varying between −80 °C and 220 °C, respecting the time given by the protocol.

Upon completion of the test, optical microscopy was performed to characterize surface morphology. Adhesion tests (Pull-off test) were conducted to quantify the effects of thermal shock on the film’s comparative mechanical integrity and mechanical integrity.

## 3. Results and Discussions

### 3.1. Contact Angle Measurements:

**Figure 5:**
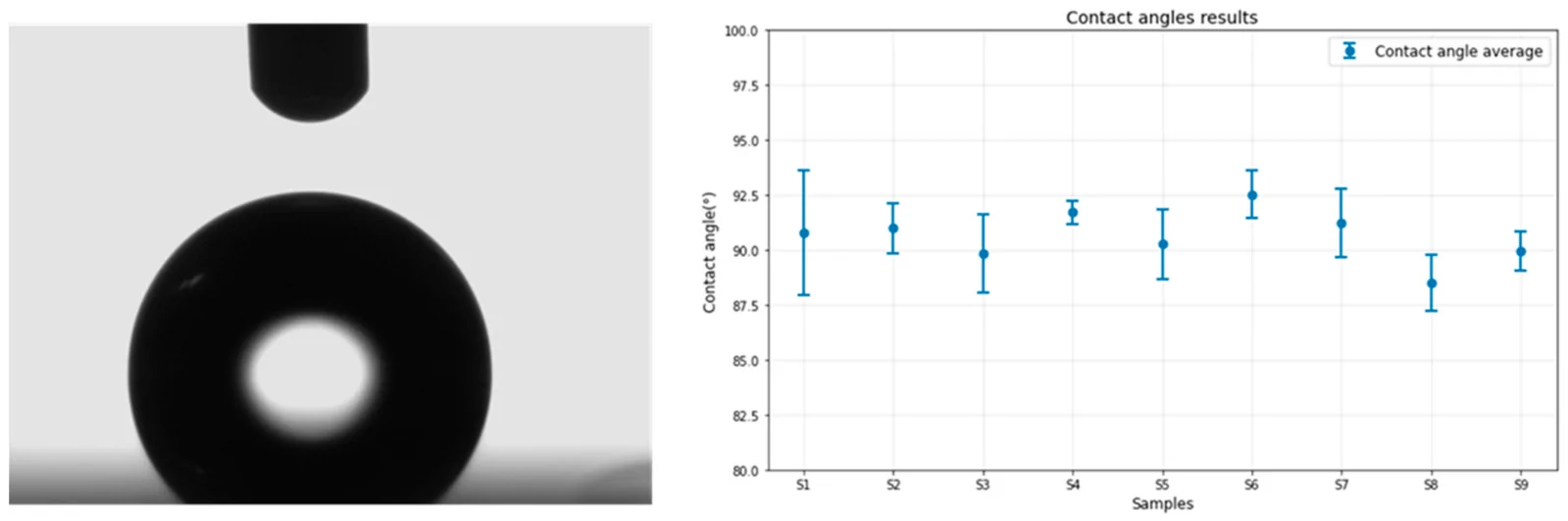
Contact angle results: parylene C on Titanium.

The contact angle measurement system provides valuable insight into the surface properties of parylene C, particularly its hydrophobicity. Figure 5 illustrates a snapshot taken by the camera the moment the water droplet contacts the sample surface and the angle measurements from all nine samples are plotted on the graph below.

**Figure 6:**
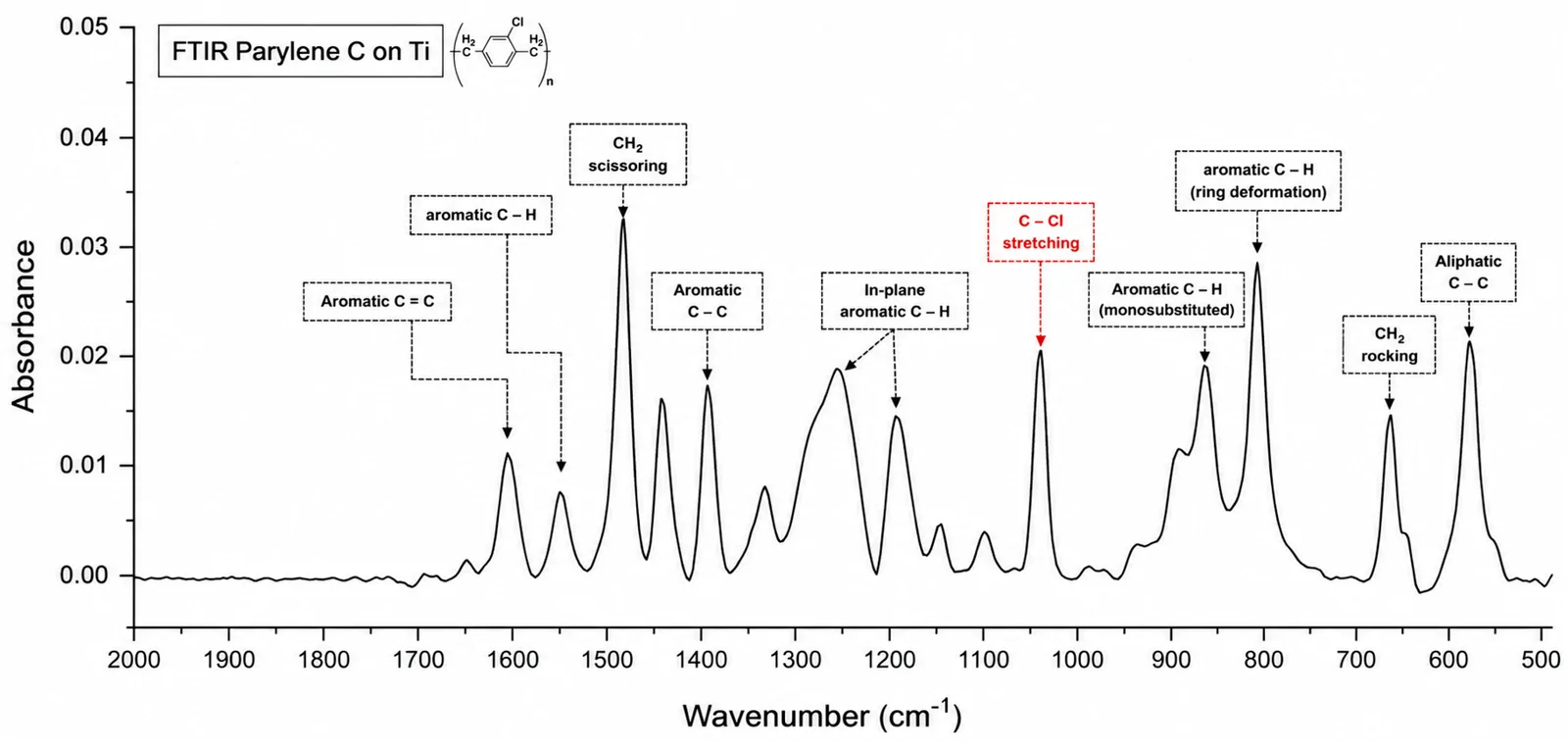
FTIR results of parylene C layer after subtracting the titanium substrate background.

A challenge encountered during the wettability characterization of parylene C was the transparency of the parylene layer on the titanium substrate. This transparency resulted in the complete reflection of the water droplet, complicating the determination of the separation between the droplet and its reflection, as depicted in Figure 6. Despite this issue, a standard limitation was established to ensure consistency across all measurements. The results confirm that the measured contact angles for all nine samples fall within a narrow range of approximately 90.56° ± 1.86 (standard deviation), consistent with the theoretical value of 90°, and in agreement with previous research [30] (CA = 91°). The low standard deviation across samples testifies to the excellent homogeneity and conformity of the parylene C layers, as well as the refinement of the deposition process.

The hydrophobicity is attributed to the presence of numerous hydrophobic aromatic rings within the parylene C coating, as revealed in the IR spectra presented in the FTIR results. These rings contribute to the material’s resistance to water, preventing wetting and promoting droplet formation on the surface, thereby confirming the hydrophobic nature of the parylene coating.

### 3.2. Chemical Composition: FTIR Results

The FTIR spectrum of the parylene C films deposited on titanium substrates (Figure 6) shows well-resolved absorption bands consistent with the molecular structure of poly(chloro-p-xylylene). The chemical structure of parylene C, (C_8_H_7_Cl)_n_, includes an aromatic benzene ring, aliphatic –CH_2_– bridges, and a chlorine substituent on the ring, which contribute to the distinct infrared absorptions observed.

A distinct absorption band at 1607 cm^−1^ is assigned to aromatic C=C stretching vibrations, representative of the benzene rings within the polymer chain. Additional peaks at 1500 and 1450 cm^−1^ are attributed to CH_2_ scissoring and tri-substituted benzene ring vibrations, while the band at 1403 cm^−1^ corresponds to aromatic C–C stretching, often associated with strain-induced vibrations in the polymer ring system. The 1200 cm^−1^ band marks in-plane aromatic C–H bending, reflecting the regular substitution pattern around the aromatic rings.

The presence of the chlorine substituent specific to parylene C is clearly confirmed by the sharp absorption at 1050 cm^−1^, assigned to the C–Cl stretching vibration. This is a key spectral signature distinguishing parylene C from its unsubstituted or fluorinated analogs.

Below 1000 cm^−1^, the peaks become increasingly complex and structurally informative. The absorption band at 876 cm^−1^ is associated with a monosubstituted aromatic C–H out-of-plane bending mode. The peak at 838 cm^−1^ also corresponds to intramolecular C–H bending within the aromatic structure. Additionally, the CH_2_ rocking and aliphatic C–C vibrations, evident at 700 cm^−1^ and 600 cm^−1,^ respectively, further support the expected polymeric methylene structure between aromatic units.

The presented FTIR spectrum is obtained after subtraction of the titanium background spectrum, which was used as a reference. As a result, the observed absorption bands are exclusive to the parylene C layer. This approach allows for direct comparison with previous studies regardless of the substrates they employed.

Overall, the FTIR spectrum supports the chemical identity of the deposited material as parylene C, while also indicating that the deposition conditions allow possible preservation of the polymer backbone, which may influence the film’s mechanical or barrier properties. These findings are consistent with prior literature [31,32] and validate the spectral sensitivity to both backbone integrity and process efficiency.

### 3.3. Chemical Composition: EDX Results

**Figure 7:**
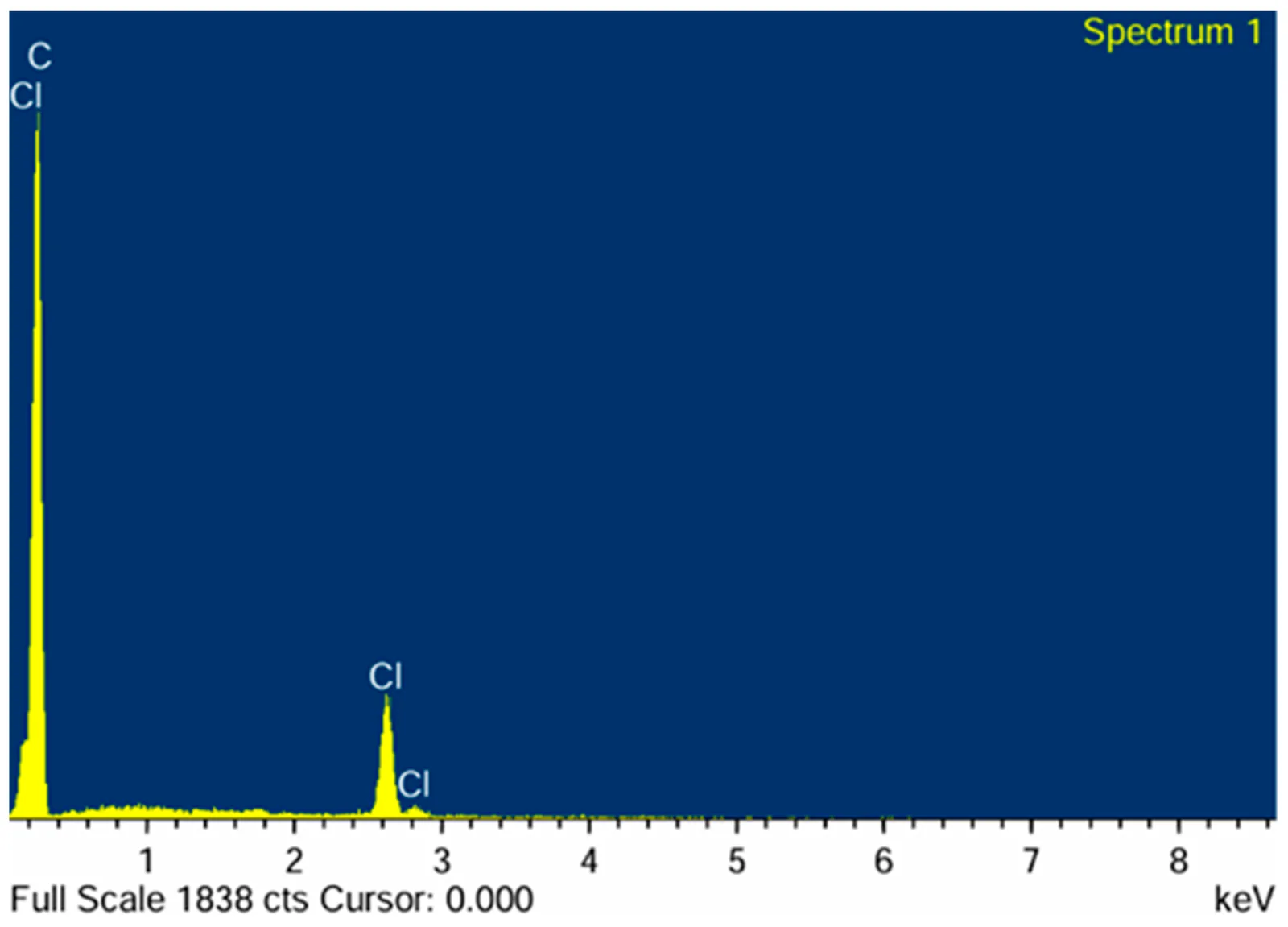
EDX Analysis of parylene C deposited on a Titanium sample.

EDX was used to confirm the chemical components present on the surface of the parylene C samples and to validate the hypothesis regarding the quantitative conformity of the atomic percentage of components. It should nevertheless be noted that EDX provides only semi-quantitative elemental information. Since hydrogen cannot be detected and the interaction volume extends beyond the outermost surface, the results should be interpreted as confirming the presence and homogeneous distribution of the characteristic detectable elements (C and Cl), rather than the exact molecular stoichiometry. According to the theory parylene C consists of the repetition of the monomer unit C_8_H_7_Cl, which means that, stochastically, the atomic percentages of C, H, and Cl should theoretically follow a ratio of 8:7:1. However, it is important to note that hydrogen atoms, being very light, are not easily detected by EDX [33], leading to a distribution of the atomic percentages. Figure 7 presents the atomic percentages for each element across the analysed sample.

Despite this, the observed results remain consistent with theoretical stoichiometry, with the proportion of carbon atoms (stable at 95.4% ± 0.19%) consistently higher than that of chlorine atoms (stable at 4.6% ± 0.19%). The low standard deviations for both elements indicate excellent chemical homogeneity and conformity of the parylene C film, reflecting a well-controlled and reproducible deposition process. These findings are consistent with prior literature [34] and confirm the chemical components present on the surface of the parylene C samples.

### 3.4. Thickness Measurement Results

The thickness of the deposited parylene C layers was analyzed using three complementary optical techniques: reflectometry, ellipsometry, and interferometry. The figures present the mean thickness and standard deviation of all measured values for samples within the different batches.

#### 3.4.1. Reflectometry Measurements

**Figure 8:**
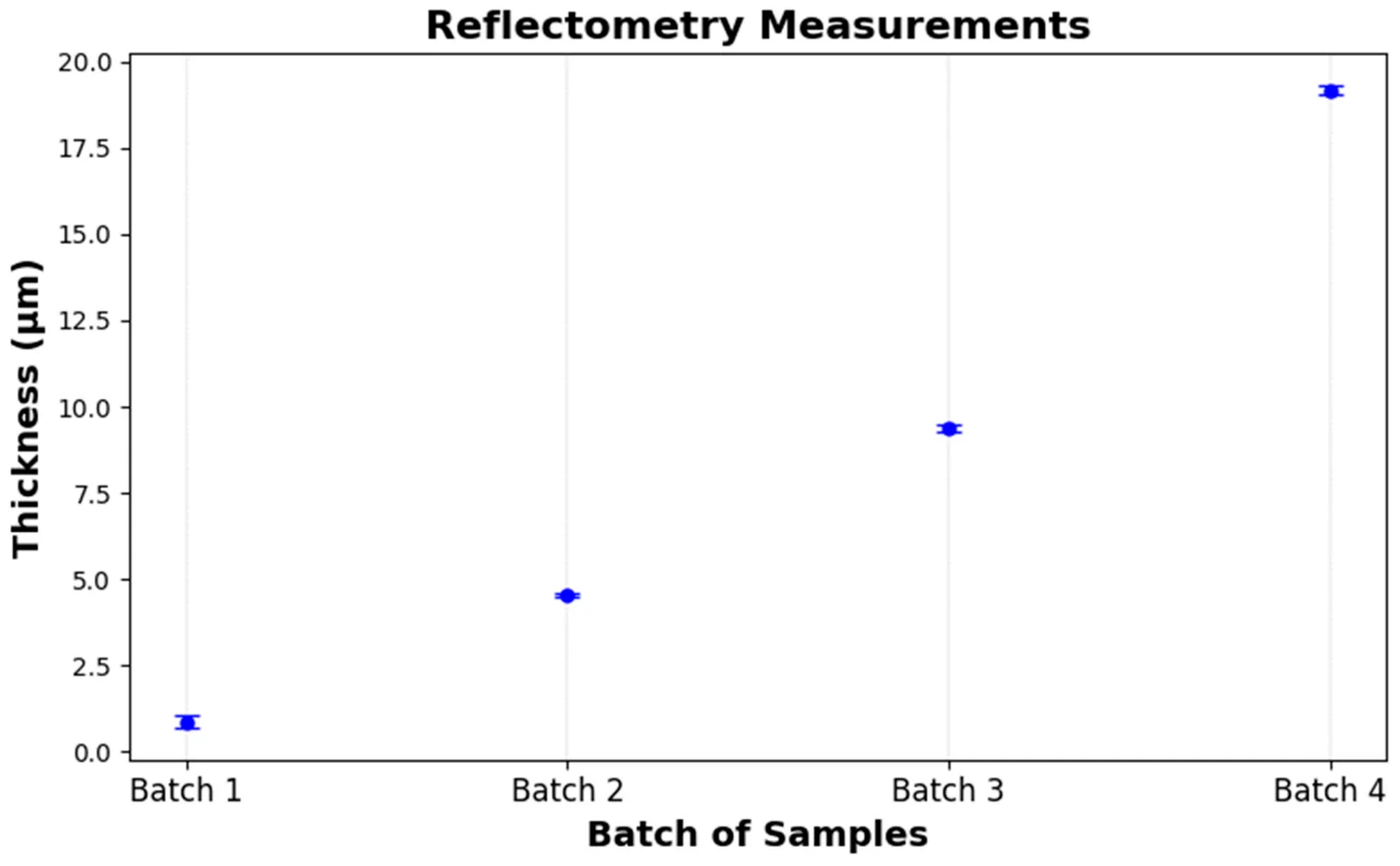
Thickness measurement results using reflectometry.

Reflectometry measurements performed on the four batches demonstrated a highly controlled and repeatable growth in film thickness, with mean values closely matching the intended targets: 0.87 µm (target: 1 µm), 4.55 µm (5 µm), 9.38 µm (10 µm), and 19.17 µm (20 µm). Intra-batch variability remained low (standard deviations ≤ 0.17 µm), reflecting excellent uniformity (Table 1). The slightly elevated dispersion observed in the thinnest layer (Batch 1, σ = 0.17 µm) is consistent with the enhanced sensitivity to surface irregularities inherent to early-stage film growth on titanium substrates (Figure 8). Altogether, these findings confirm that the deposition process is both stable and scalable across the targeted thickness range.

#### 3.4.2. Ellipsometry Measurements

**Figure 9:**
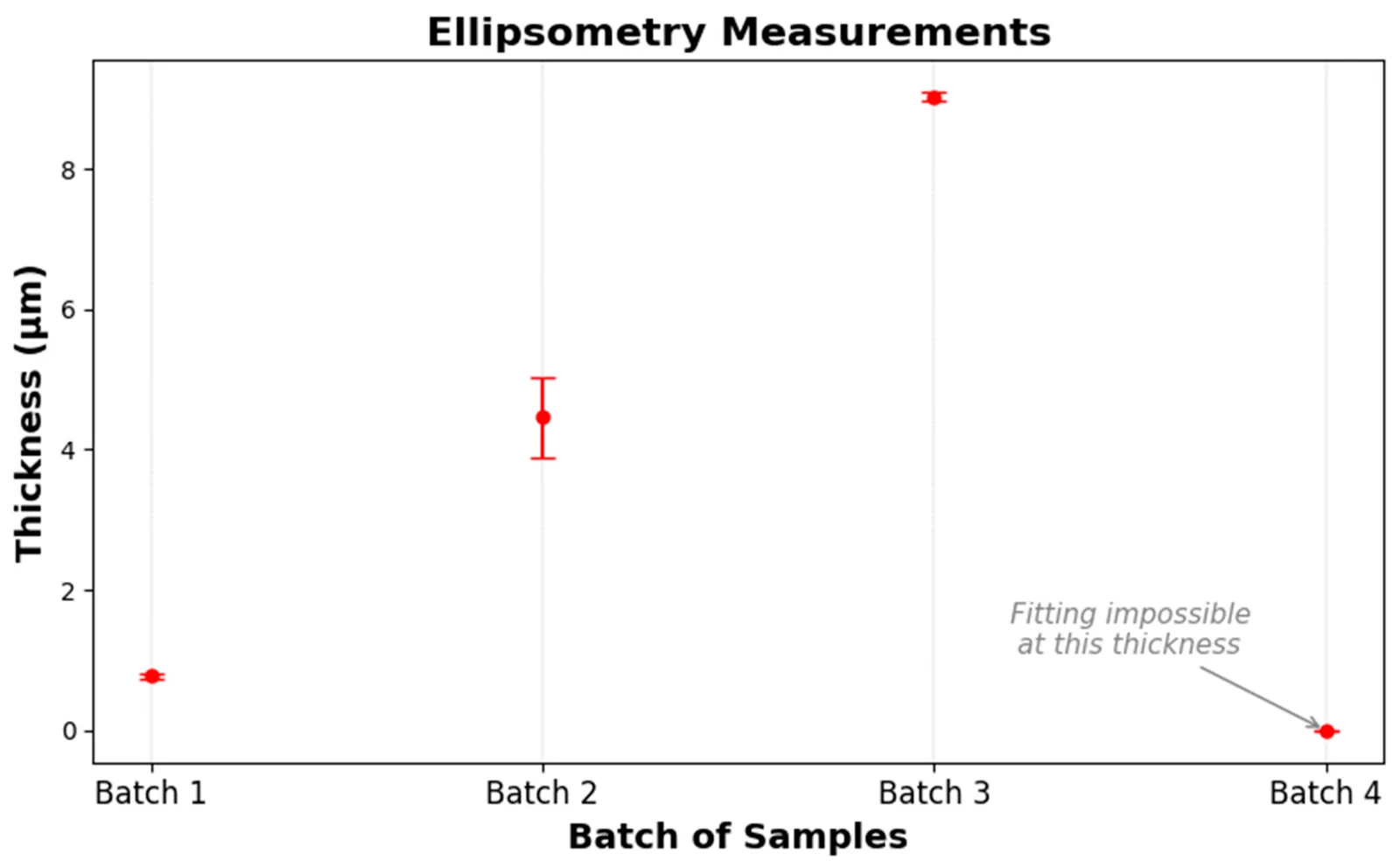
Thickness measurement results using ellipsometry.

Ellipsometry analysis of the same four deposition batches yielded mean thicknesses of 0.78 µm (σ = 0.04 µm), 4.46 µm (σ = 0.58 µm), and 9.03 µm (σ = 0.06 µm) for Batches 1 to 3, respectively (Table 2). The relatively low dispersion in Batches 1 and 3 underscores the technique’s precision in thin- and moderate-thickness regimes, while the larger standard deviation observed in Batch 2 (σ = 0.58 µm) suggests greater fitting uncertainty around 5 µm (Figure 9).

No reliable ellipsometric fit was obtained for Batch 4 (target 20 µm), indicating that the film exceeded the measurement range and/or model validity for the instrument configuration. Overall, ellipsometry provides accurate and reproducible thickness data up to approximately 10 µm but requires alternative approaches or extended modeling for films beyond this limit.

#### 3.4.3. Interferometry Measurements

**Figure 10:**
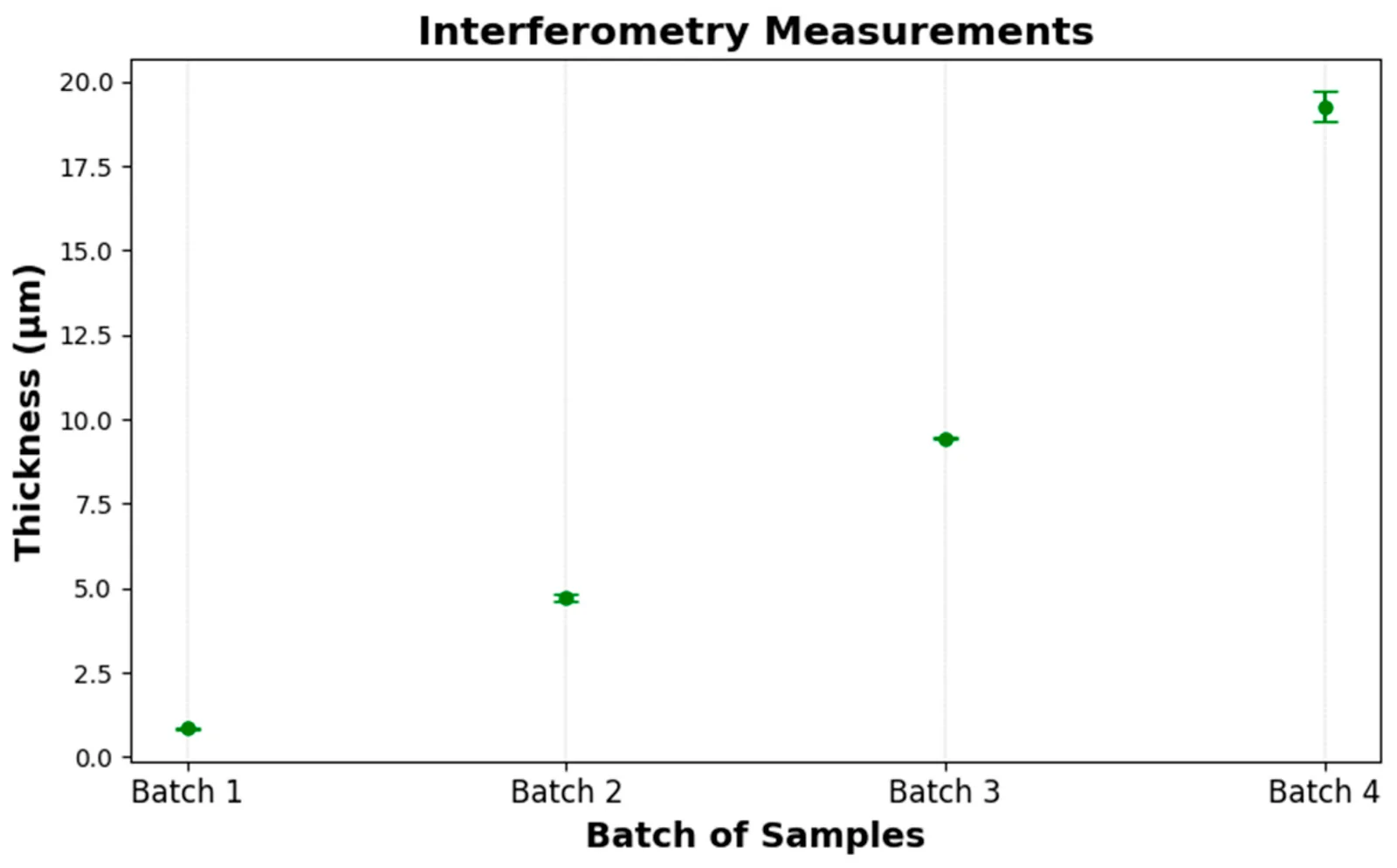
Thickness measurement results using interferometry.

Interferometry determined mean thicknesses of 0.85 µm (σ = 0.03), 4.70 µm (σ = 0.10), 9.44 µm (σ = 0.03), and 19.26 µm (σ = 0.45) for Batches 1 to 4, respectively. These values closely match the targets (1, 5, 10, 20 µm), demonstrating the method’s accuracy across the full range (Table 3). Precision is highest in the thinnest and mid-range films (σ ≤ 0.10 µm) and decreases slightly for the thickest layer (σ = 0.45 µm), where interference fringes become more closely spaced. Overall, interferometry provides reliable, repeatable thickness measurements from 1 µm up to about 20 µm (Figure 10).

**Figure 11:**
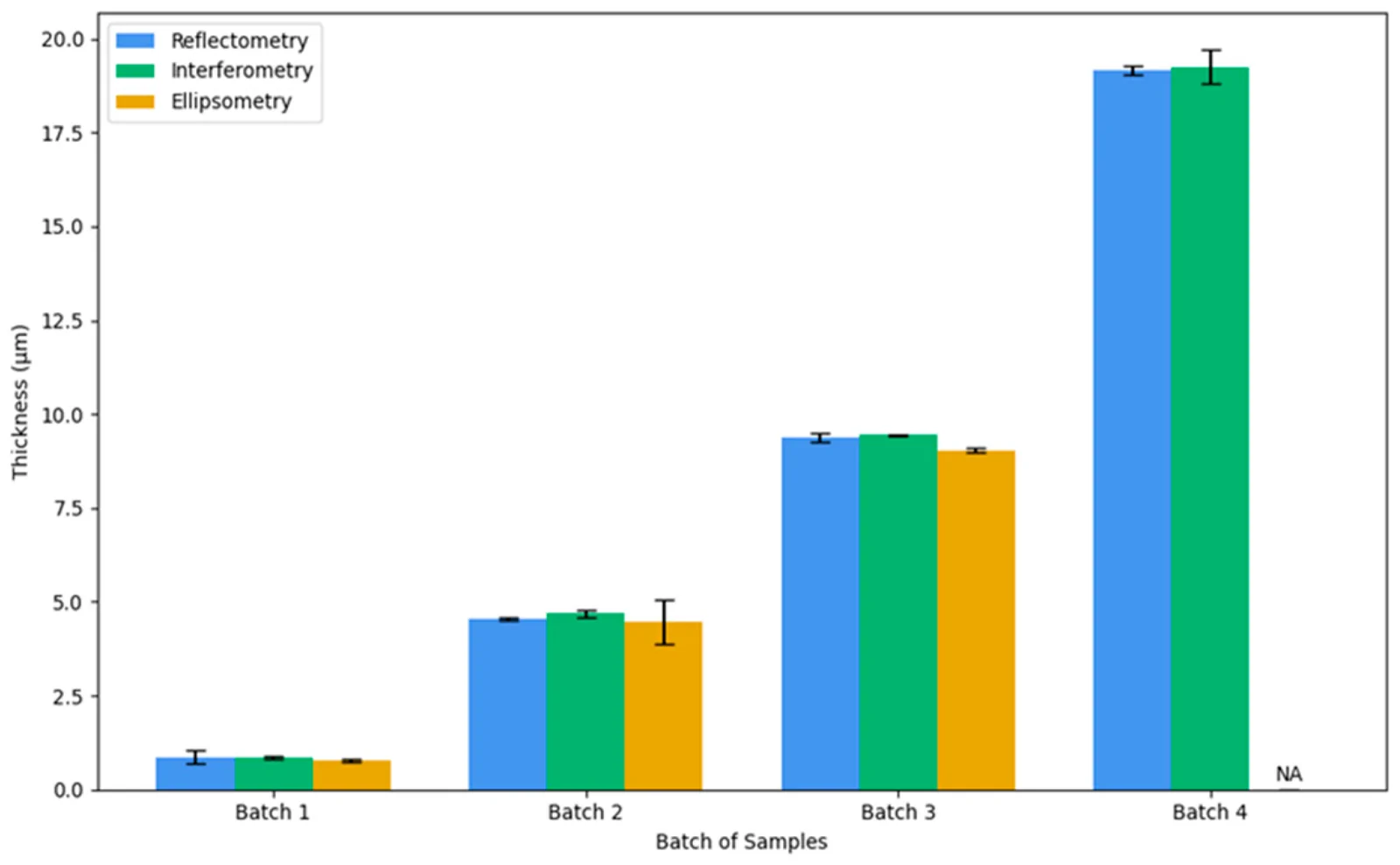
Superposition of measurements across all batches using three different techniques.

Figure 11 illustrates the superposition of measurements conducted on all samples across all batches using three different techniques. From it, we can deduce that the measurements exhibit nearly identical thickness values, with a minimal disparity of approximately 0.2 µm. Some difficulties were encountered in determining the thickness of the fourth batch (20 µm) via ellipsometry due to the technique’s thickness measurement limitation.

Furthermore, it is evident that all three measurement methods yield more consistent results for thinner films, as reflected by the lower standard deviation values in the initial batches. However, as the coating thickness increases, notably in Batch 4, the standard deviation becomes larger, indicating greater variability between methods and potentially reduced precision for thicker films.

### 3.5. Roughness Analysis (AFM)

**Figure 12:**
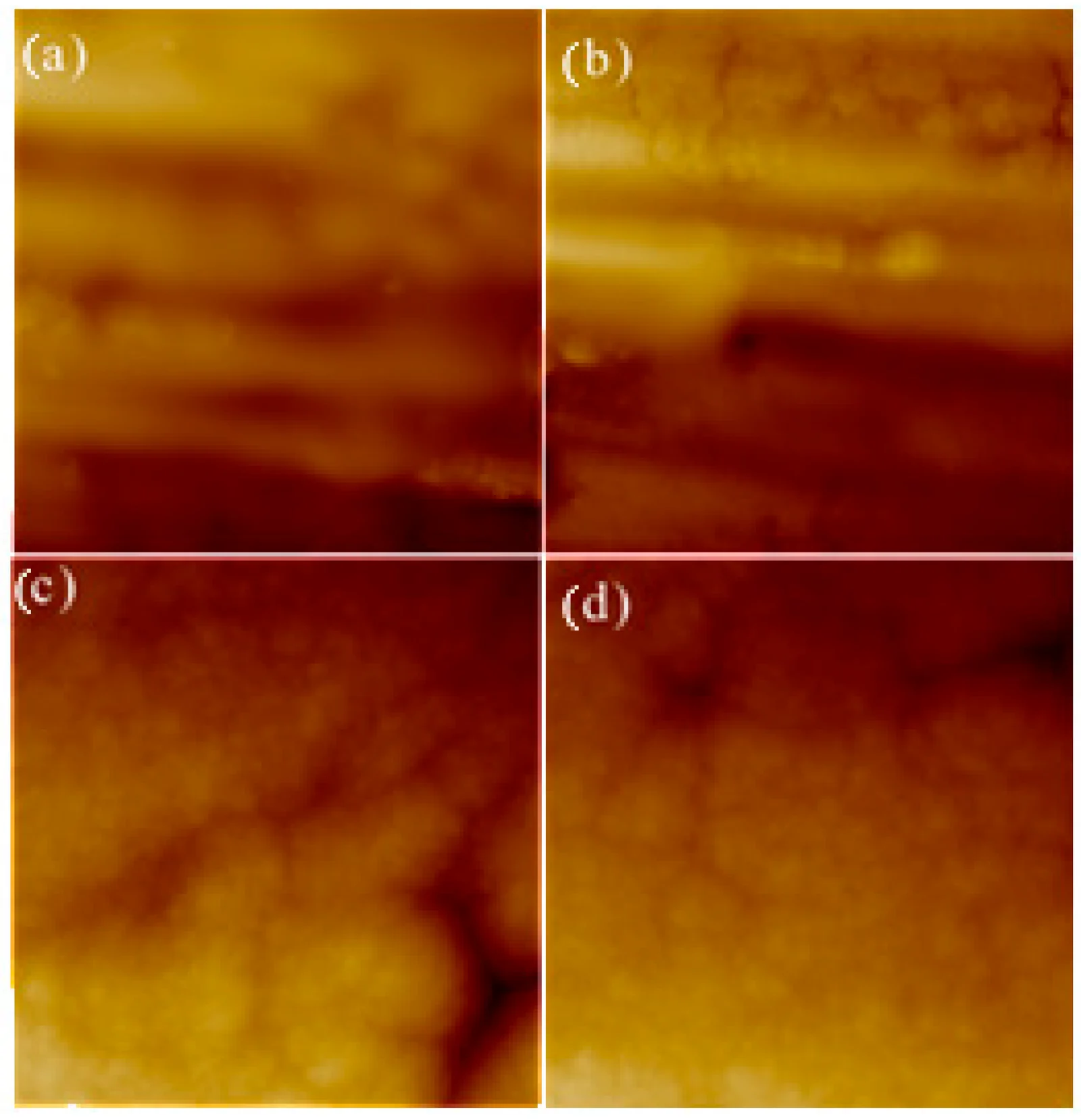
AFM surface roughness analysis: (**a**,**b**) AFM images of the uncoated titanium sample, showing a mean Ra of 91.7 ± 20.7 nm. (**c**,**d**) AFM images of parylene C on titanium samples (5 µm thickness–10 × 10 µm^2^), with a mean Ra of 93.7 ± 20.4 nm.

The results indicate that the average roughness (Ra) of the parylene C films is approximately 94 nm, with a standard deviation of about 20 nm. This relatively high variation is attributed to the unpolished titanium wafers used as substrates, which themselves exhibit a similar average Ra of approximately 92 nm with comparable standard deviation (Figure 12). These findings suggest that the parylene C films closely conform to the underlying titanium surface morphology, resulting in a roughness that mirrors that of the uncoated titanium substrate [35].

### 3.6. Application Based Characterization Results

#### 3.6.1. Adhesion Pull-Off Test Results for As-Deposited Parylene C Films

**Figure 13:**
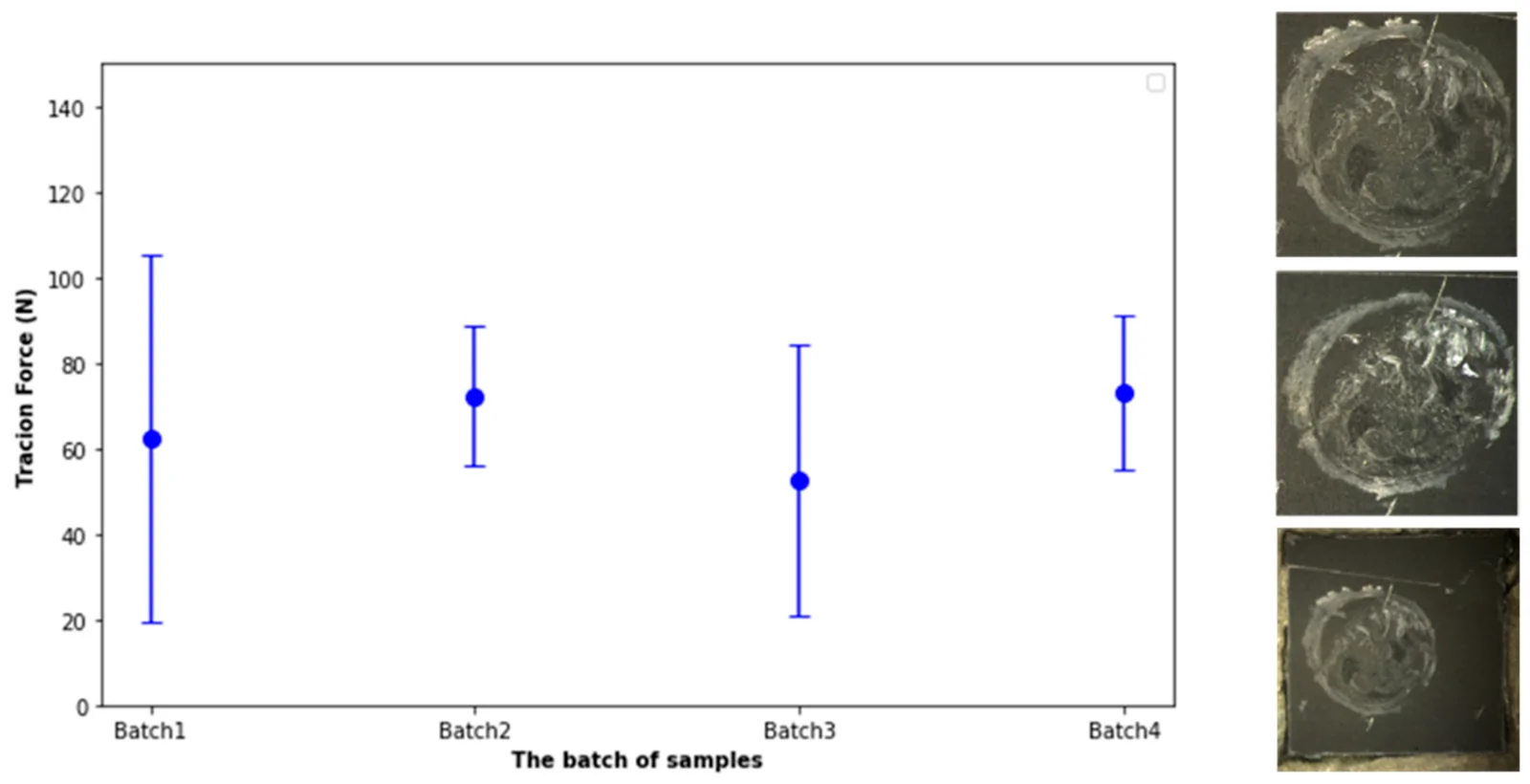
Pull test results per batch and post-test observations.

The pull-off configuration employed in this study was intended primarily to compare the mechanical integrity of identical coating systems before and after environmental ageing. Because failure occurred within the adhesive rather than at the coating/substrate interface, the measured force should not be interpreted as the intrinsic interfacial adhesion energy. Standardized pull-off methods such as ASTM D4541 and ISO 4624 provide recommendations for quantitative adhesion measurements [36,37]. Figure 13 presents the relationship between the applied detachment force and the different batches of parylene C films on titanium. Each data point represents the average force value for a batch, along with its standard deviation, demonstrating the reproducibility of pull tests across samples. These results are further detailed in Table 4, which sums up the average traction forces and standard deviations for four batches.

Batch 1 exhibited an average detachment force of 62.45 N with a high standard deviation of 42.87 N, indicating significant variability among samples, likely due to inconsistencies in adhesive application or substrate surface conditions. Batch 2 showed improved consistency, with a higher average force of 72.25 N and a lower standard deviation of 16.40 N, suggesting better uniformity in surface treatment or adhesive behaviour. Batch 3, by contrast, had the lowest average force of 52.71 N and a considerable standard deviation of 31.73 N, reflecting weaker and more scattered adhesion performance. Batch 4 demonstrated the highest average detachment force at 73.23 N with a relatively moderate standard deviation of 18.06 N, making it the most reliable batch in terms of both strength and repeatability. In all cases, detachment occurred due to the failure of the adhesive, rather than the bond between the parylene C film and the titanium substrate. The maximum force recorded in each test corresponds to the adhesive’s holding capacity before breaking. Although the detachment was not uniformly distributed across the entire surface of the chip in all tests, consistent behaviour was observed among samples within the same batch.

Interestingly, during subsequent multimeter tests, current flow was detected due to the conductive titanium layer beneath the parylene, confirming partial detachment of the film. Despite the overall consistency of mean traction forces across the four batches, indicating good reproducibility between batches, the high standard deviations observed within each batch reveal a low reproducibility at the intra-batch level. This suggests that while the average comparative mechanical integrity of parylene C on titanium is robust and comparable across different test groups, variations within each batch are significant.

#### 3.6.2. Corrosion Test Results

**Figure 14:**
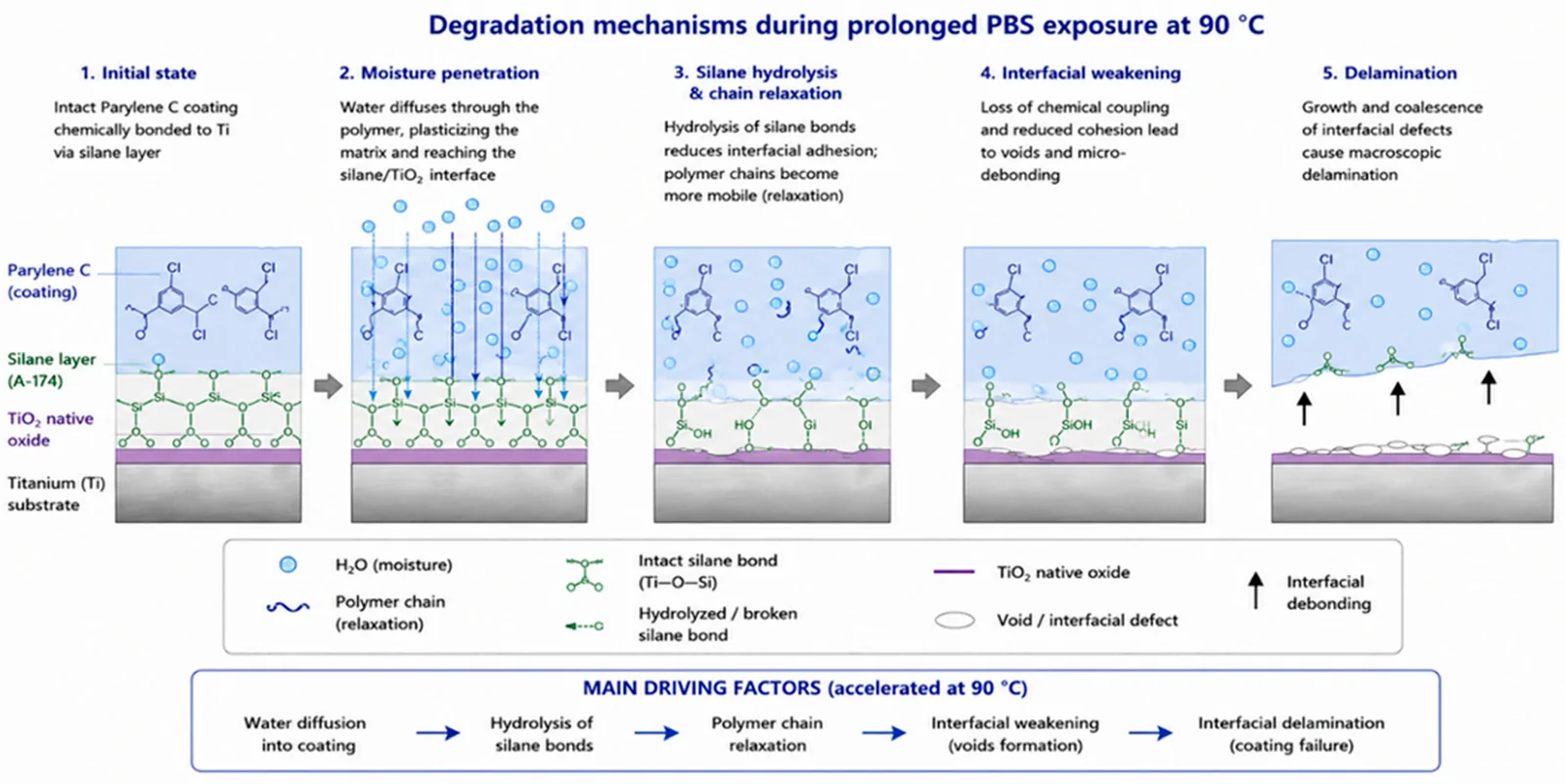
Schematic illustration of the degradation mechanisms occurring during prolonged PBS exposure at 90 °C. Water diffusion through the Parylene C coating progressively weakens the silane coupling layer by hydrolysis, promotes polymer chain relaxation, generates interfacial defects and ultimately leads to coating delamination.

To facilitate the interpretation of the experimental observations, Figure 14 schematically illustrates the degradation mechanisms expected during prolonged PBS exposure at elevated temperature. Water progressively diffuses through the Parylene C coating and reaches the silane coupling layer, where hydrolysis weakens the Ti–O–Si bonds. Simultaneously, moisture-induced polymer chain relaxation reduces the mechanical integrity of the coating, promoting interfacial void formation and progressive delamination. These mechanisms are consistent with the reduction in pull-off force observed after accelerated ageing.

**Figure 15:**
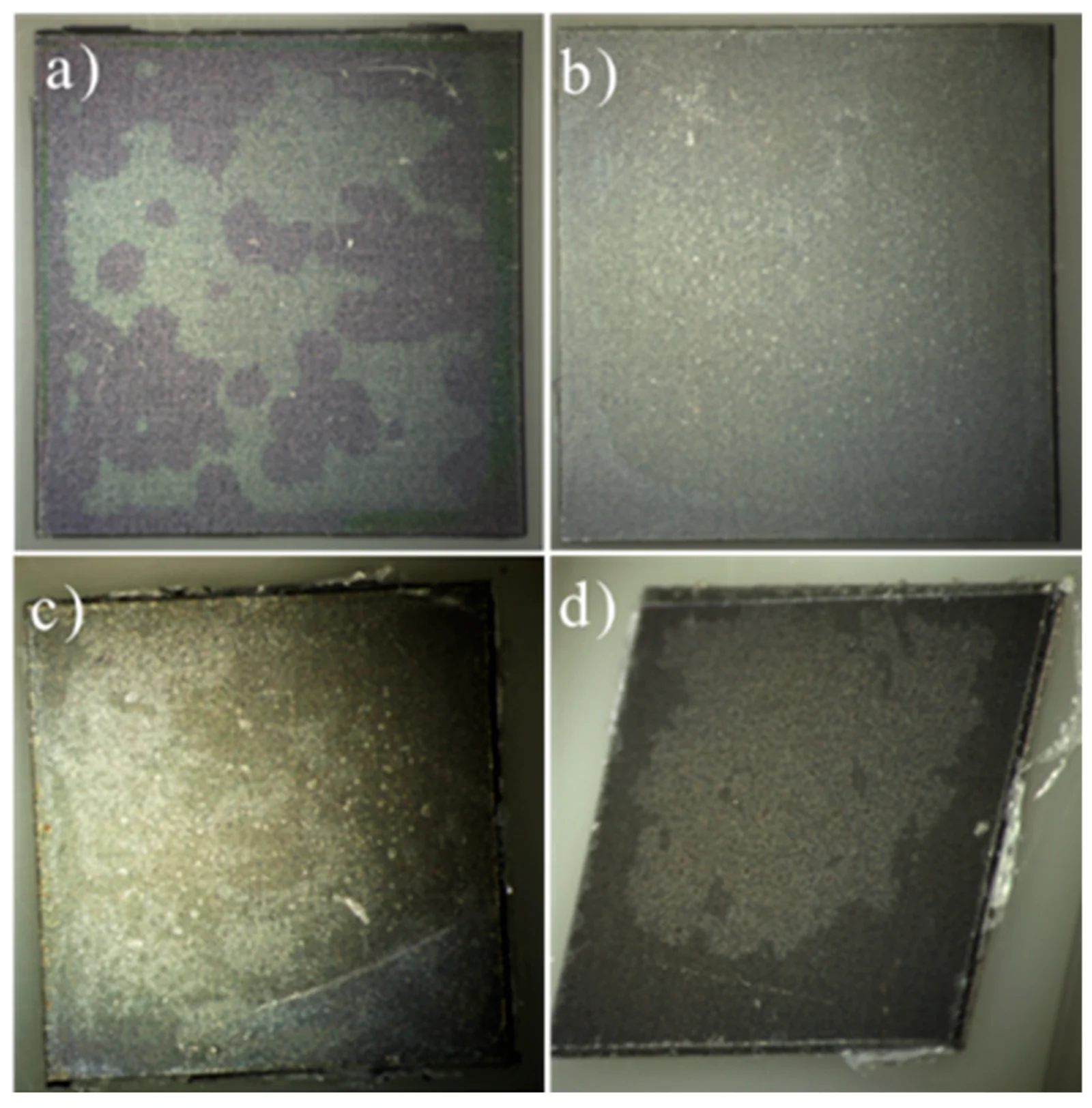
Microscopic images of batches (**a**) 1 µm, (**b**) 5 µm, (**c**) 10 µm, and (**d**) 20 µm parylene C coatings after the corrosion test (90 °C). The visual comparison highlights the surface degradation and delamination performance across different coating thicknesses.

After the accelerated PBS ageing test, consisting of immersion in a saline solution at 90 °C for 21 days, significant peeling and delamination of the parylene layer were observed across several areas of the samples (Figure 15). This degradation may be attributed to multiple factors. One possibility is the failure of the adhesion promoter (A-174 silane), which may not have maintained its bonding properties under prolonged exposure to high temperature and aggressive chemical conditions. Another contributing factor could be the parylene layer itself, as parylene C has a continuous use temperature (CUT) limited to approximately 80 °C [38]. Exposure to temperatures exceeding this threshold over an extended period can lead to polymer chain scission, reduction in mechanical integrity, and loss of adhesion to the substrate. These results suggest that both the stability of the adhesion promoter and the thermal endurance of the parylene layer must be carefully considered when designing protective coatings for harsh environments [13].

**Figure 16:**
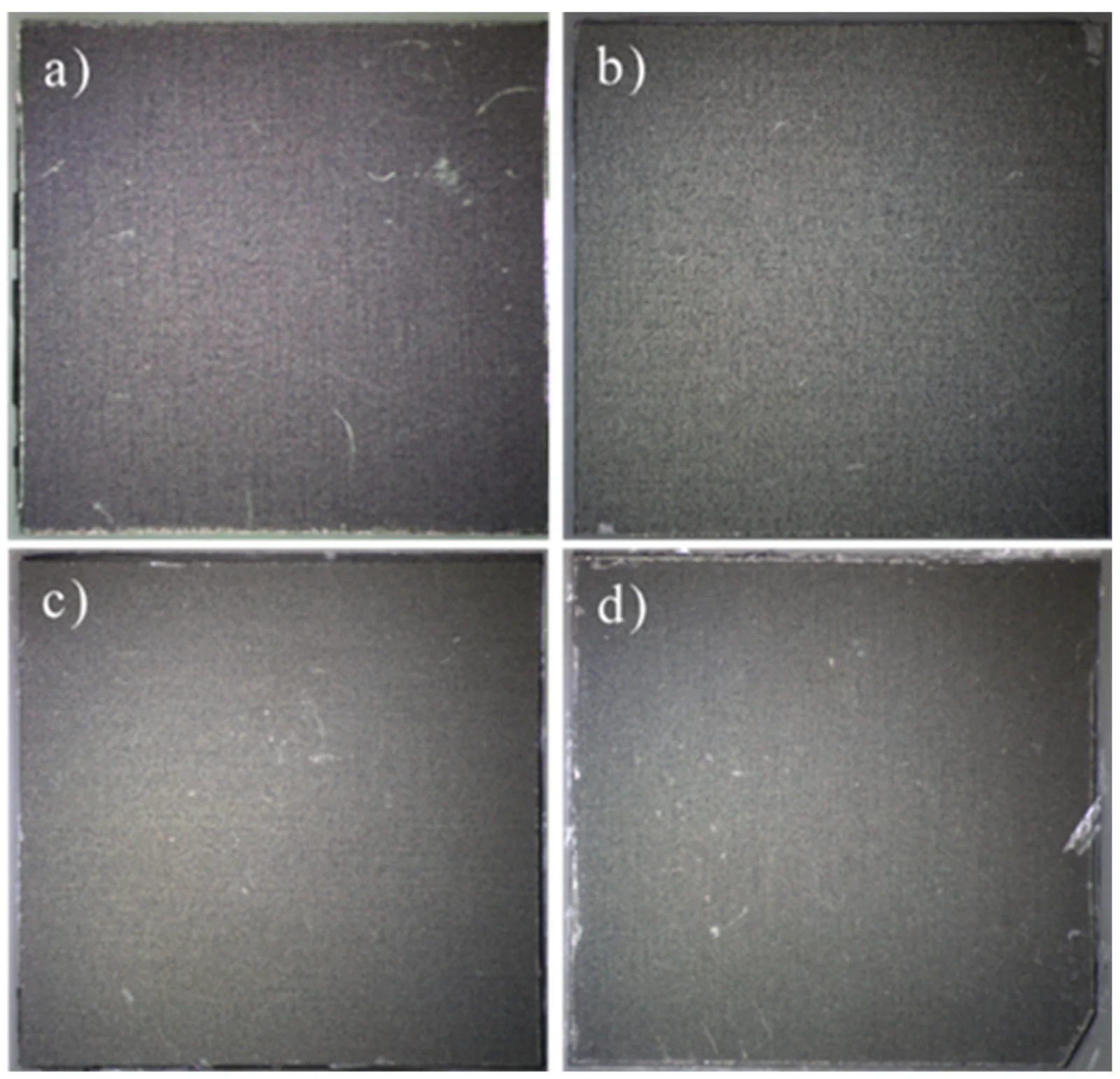
Microscopic images of control samples (**a**) 1 µm, (**b**) 5 µm, (**c**) 10 µm, and (**d**) 20 µm Parylene C coatings after 21 days of immersion in saline at room temperature. No visible degradation or delamination is observed, confirming the critical role of elevated temperature in the corrosion-induced failure of the coatings.

The control samples from each batch were maintained at room temperature for the duration of the accelerated PBS ageing test and serve as negative controls for thermal and chemical exposure. Figure 16 shows microscopic images of these control samples, coatings with mean thicknesses of 1 µm, 5 µm, 10 µm, and 20 µm, after 21 days in saline at ambient temperature.

In contrast to the pronounced peeling and delamination observed in samples subjected to 90 °C, the room-temperature controls display continuous, defect-free parylene C films with no signs of peeling, cracks, or delamination.

**Figure 17:**
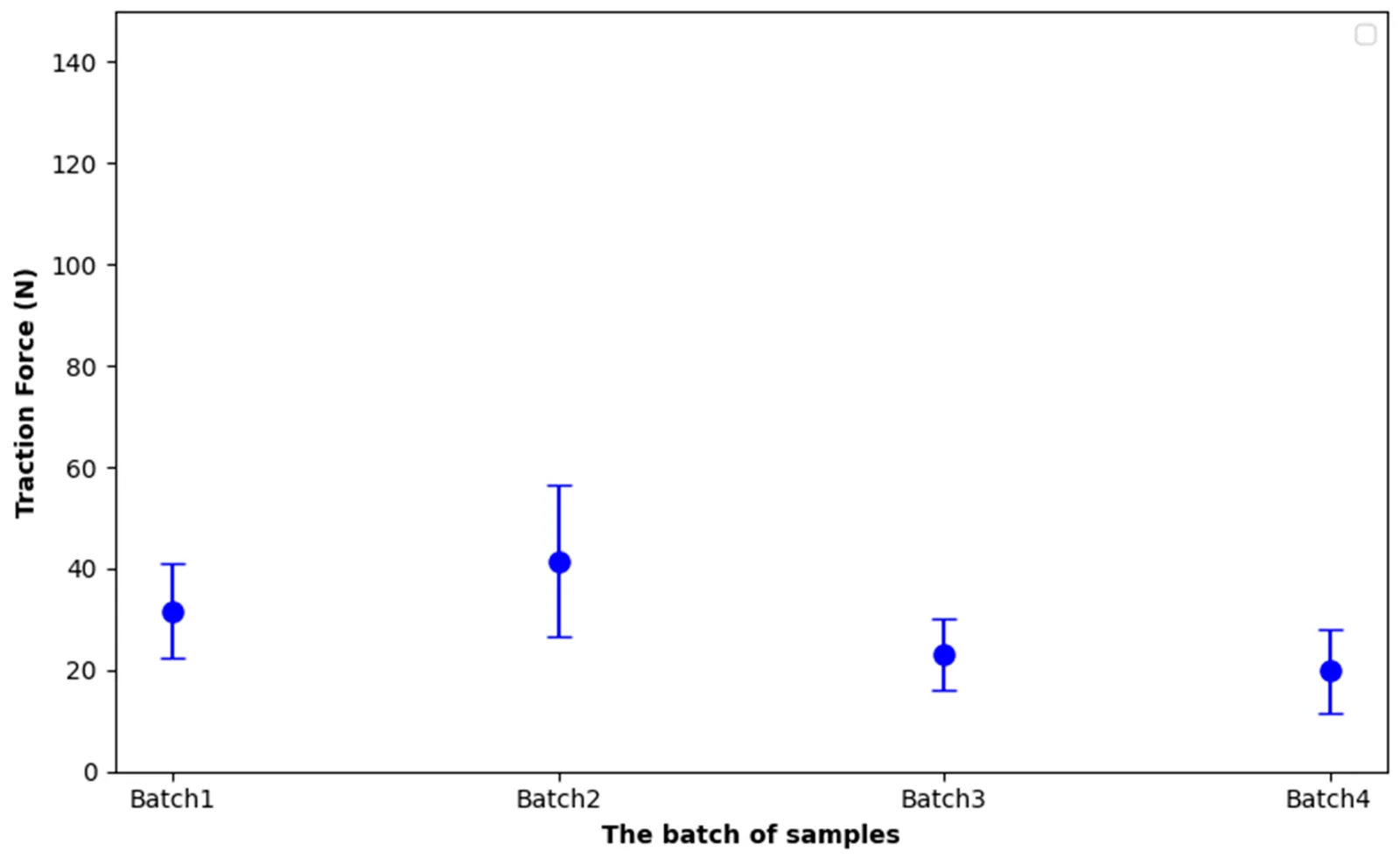
Pull test results per batch after corrosion.

**Figure 18:**
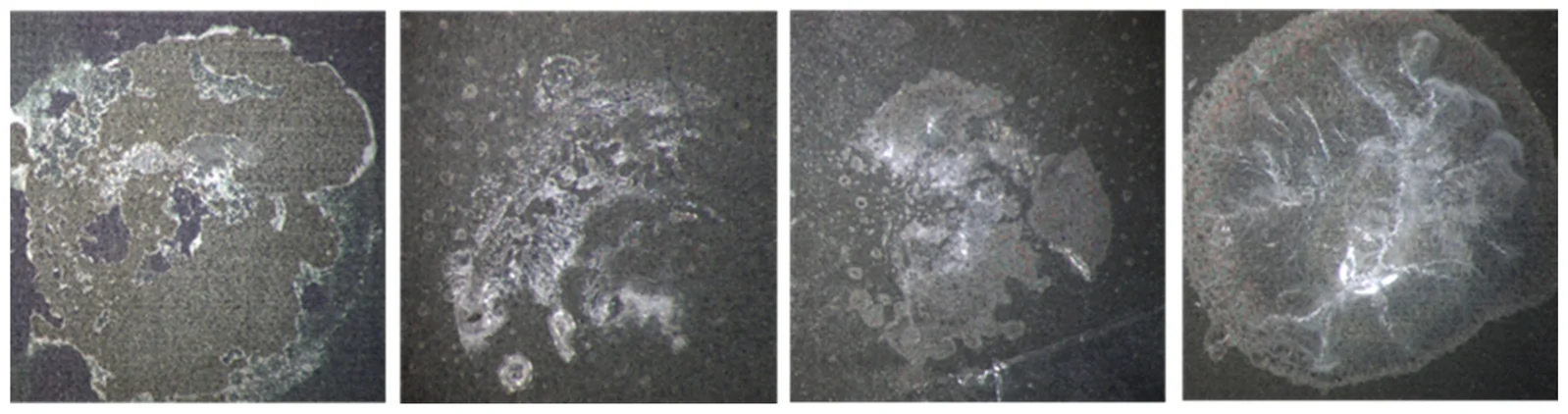
Microscopic Images of parylene C Coatings (1 µm to 20 µm) After Pull Tests Following Corrosion Exposure.

These findings confirm that the degradation mechanisms are driven primarily by elevated temperature rather than chemical attack alone and underscore the necessity of accounting parylene C’s thermal limits when specifying protective layers for environments combining corrosive media and elevated temperatures. These observations are in accord with findings reported in referenced studies [39,40], which similarly highlight the degradation of parylene’s mechanical and adhesive properties under prolonged thermal and oxidative stress. Moreover, the observations from the post-corrosion microscopic analysis need to be validated, for that reason pull-off tests were performed on four samples from each batch. The results of Figure 17, summed up in Table 5, reveal a noticeable decline in the traction force required to detach the parylene C coating from the titanium surface across the batches. Despite the increase in coating thickness from Batch 1 to Batch 4, there is a significant decrease in traction force values after the accelerated PBS ageing test, with forces reduced by approximately 45% to 60% compared to the as-deposited samples. This trend highlights the impact of corrosion-induced damage, specifically, the pre-existing delamination caused by prolonged exposure to the corrosive environment appears to compromise the interfacial adhesion. These results suggest that beyond a certain threshold, increasing parylene C thickness does not necessarily enhance adhesion if corrosion has already undermined the coating’s structural integrity (Figure 18).

#### 3.6.3. Thermal Shock Test Results

**Figure 19:**
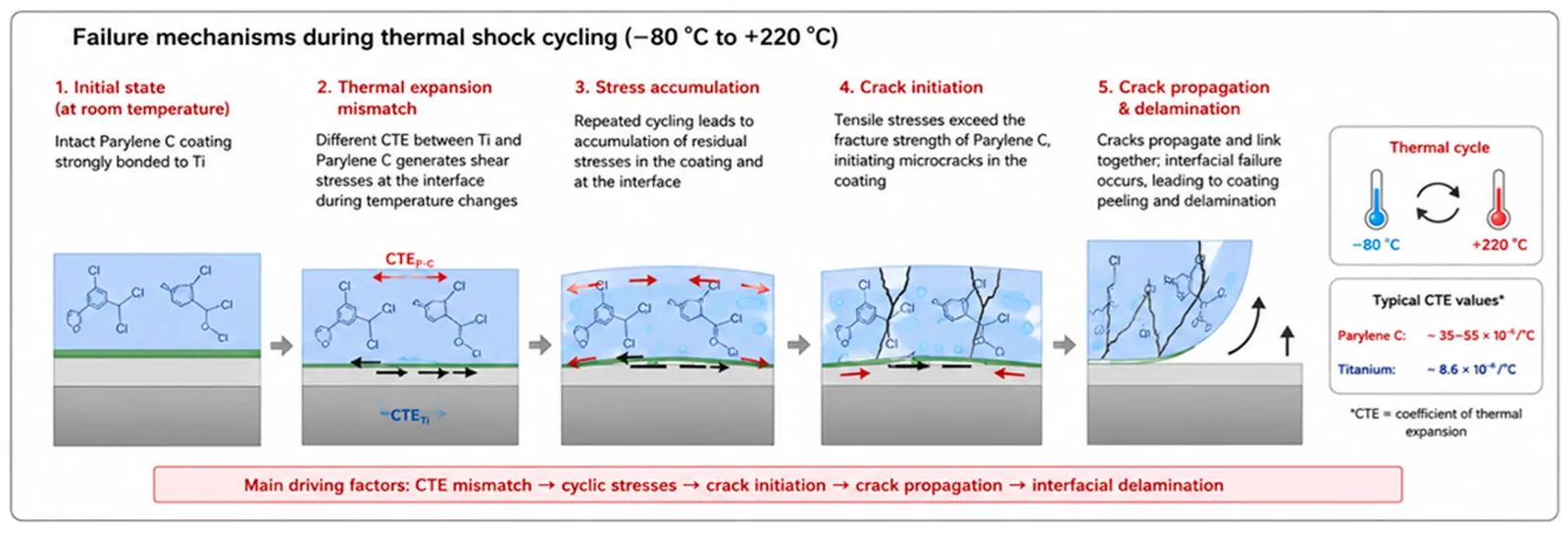
Schematic illustration of the degradation mechanisms occurring during thermal-shock cycling (−80 °C to +220 °C). The mismatch between the coefficients of thermal expansion of titanium and Parylene C generates cyclic interfacial stresses that promote crack initiation, crack propagation and progressive coating delamination.

The objective of this protocol was not to reproduce physiological conditions but to evaluate coating robustness under severe manufacturing and qualification environments, including electronic packaging, solder reflow, thermal bonding and reliability assessment. This approach is consistent with qualification procedures described in MIL-STD-202 Method 107G [29]. Figure 19 summarizes the physical mechanisms responsible for coating failure during thermal-shock cycling. Owing to the large difference in coefficients of thermal expansion between titanium and Parylene C, repeated heating and cooling generate cyclic interfacial shear stresses. These stresses progressively accumulate, leading to crack initiation within the polymer layer, crack propagation and finally interfacial delamination, in agreement with the severe degradation experimentally observed after thermal cycling.

**Figure 20:**
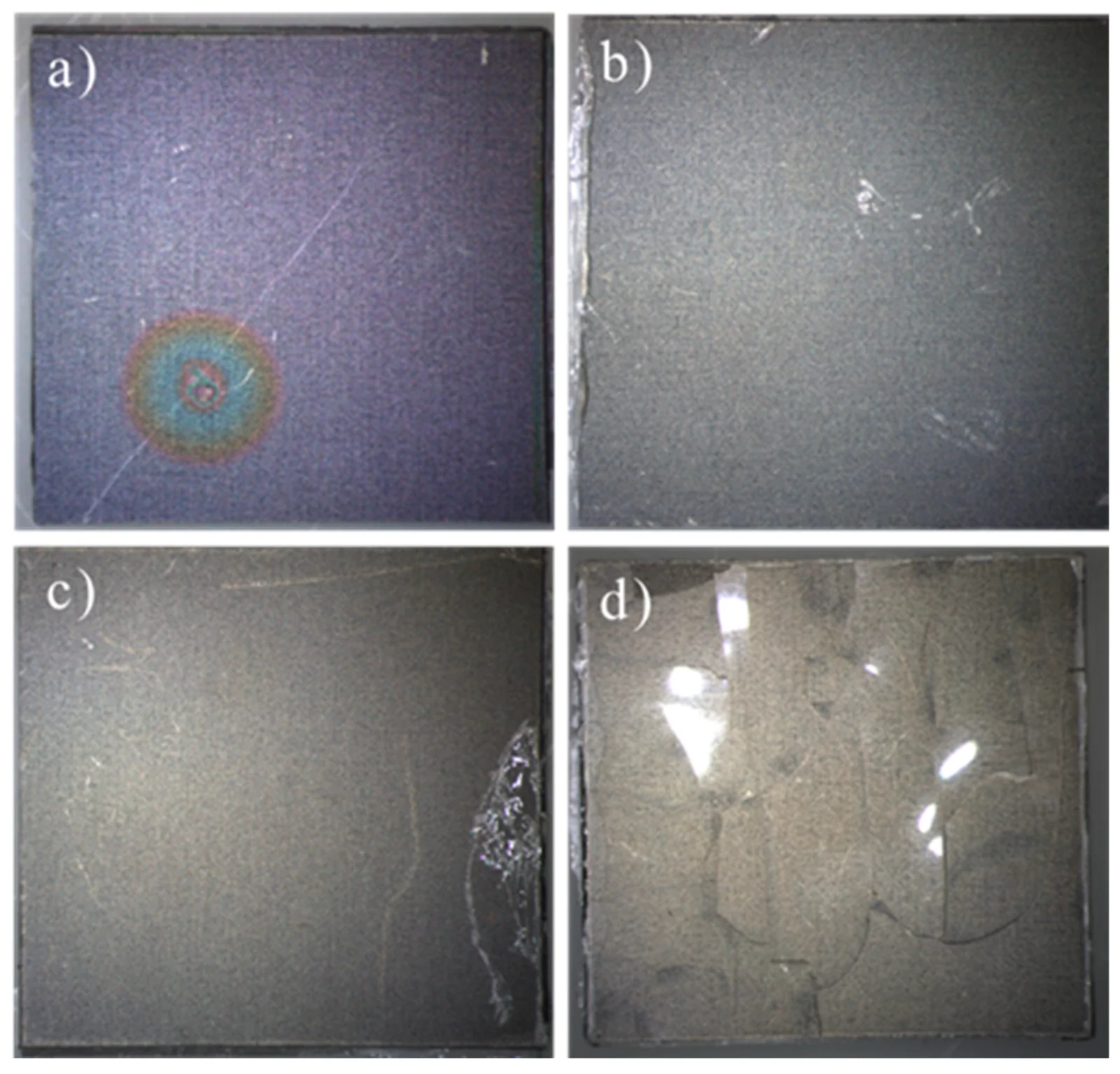
Microscopic images of batches (**a**) 1 µm, (**b**) 5 µm, (**c**) 10 µm, and (**d**) 20 µm parylene C coatings after the thermal shock test. The visual comparison highlights the surface degradation and delamination performance across different coating thicknesses.

Delamination and damage zones were observed in the parylene coatings of all batches following thermal cycling (Figure 20), which may stem from insufficient adhesion to the titanium substrates or from the extreme temperatures to which the samples were exposed. These observations are consistent with the accelerated PBS ageing test findings. In that case, failure of the A-174 silane adhesion promoter under prolonged chemical and thermal stress, and the fact that parylene C’s continuous use temperature is limited to around 80 °C, likely contributed to polymer chain scission, loss of mechanical integrity, and eventual loss of adhesion.

**Figure 21:**
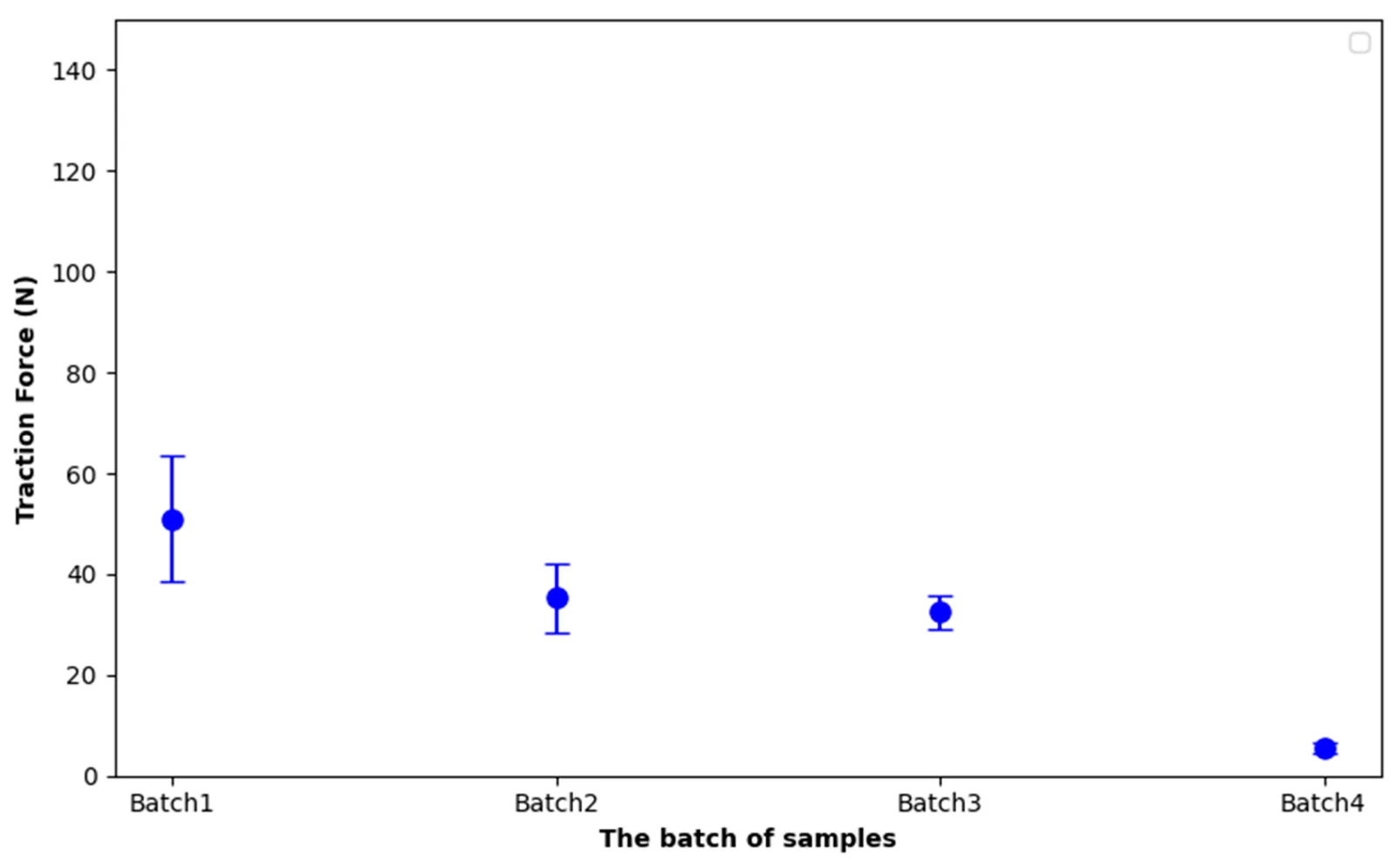
Pull test results per batch after thermal shock.

**Figure 22:**
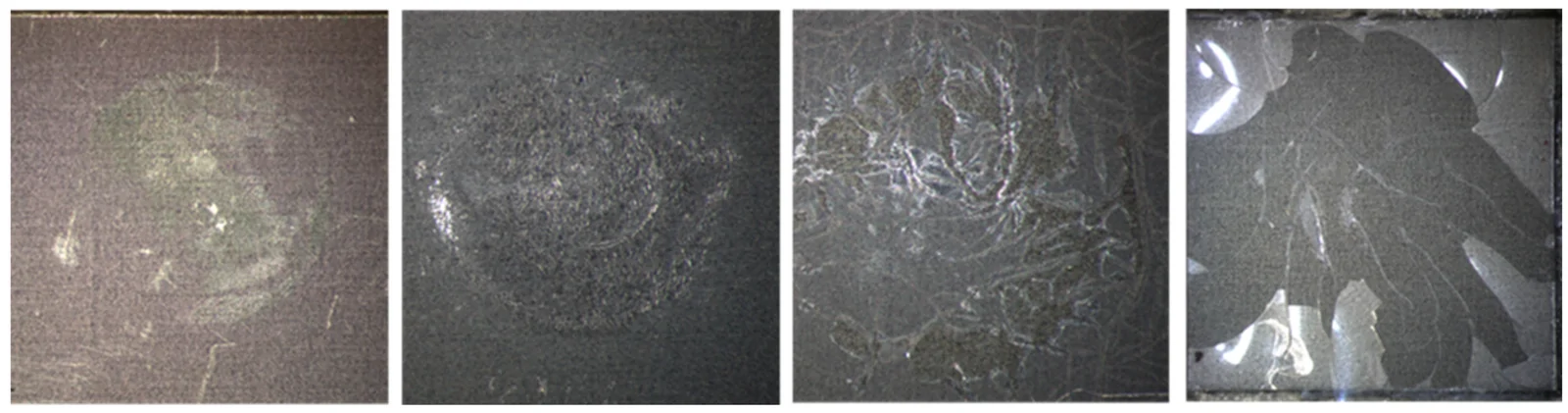
Microscopic Images of parylene C Coatings (1 µm to 20 µm) After Pull Tests Following thermal shock test.

The effects of thermal shock on adhesion performance need to be investigated, thus pull-off tests were conducted on four samples from each batch. The results of Figure 21, resumed in Table 6, show a clear degradation in comparative mechanical integrity with increasing coating thickness. Despite the increase in parylene C thickness from Batch 1 to Batch 4, the traction force required to pull off the coating significantly decreases, with Batch 4 showing a drastic drop to only 5.5 N, which corresponds to a reduction in more than 90% compared to the as-deposited condition. Overall, the forces in other batches also show a reduction of around 50% compared to the baseline. This sharp decline in Batch 4 is consistent with microscopic observation (Figure 22), which reveals that the parylene coating is almost fully delaminated from the titanium substrate after thermal shock exposure. The thermal stress likely induced microcracks and interfacial damage that compromised adhesion.

These findings suggest that increasing the coating thickness beyond a certain point may not improve adhesion performance under rapid temperature cycling, especially if the coating integrity is already compromised by thermal mismatch or interfacial stress. And they underscore the need to optimize both adhesion chemistry and the thermal endurance of parylene coatings for reliable performance in harsh environments.

## 4. Conclusions

In this work, parylene C coatings of four target thicknesses (1 µm, 5 µm, 10 µm, and 20 µm) were conformally deposited onto grade 1 titanium substrates and subjected to a comprehensive suite of chemical, morphological, mechanical, and environmental tests. Surface characterization by contact angle goniometry and AFM revealed consistently hydrophobic, smooth films whose roughness closely followed the underlying titanium topography. FTIR and EDX confirmed the expected chemical structure, aromatic C=C, C–H, and C–Cl vibrations in the infrared spectrum, and carbon-to-chlorine atomic ratios matching parylene C stoichiometry.

Thickness measurements by reflectometry, ellipsometry, and interferometry demonstrated excellent control over film growth, with mean values closely matching the design targets for each batch (1 µm, 5 µm, 10 µm, and 20 µm) and minimal variation across all batches. For the 1 µm batch, standard deviations ranged from 0.03 µm to 0.17 µm, for the 5 µm batch, from 0.05 µm to 0.58 µm, for the 10 µm batch, from 0.03 µm to 0.11 µm and for the 20 µm batch, from 0.13 µm to 0.45 µm. Overall, the convergence of values and low dispersion confirm the robustness and reliability of the deposition and characterization protocols.

Pull-off adhesion tests initially indicated strong bonding between parylene C and titanium, with average detachment forces ranging from 52 N to 73 N under room-temperature conditions. However, after 21 days of accelerated PBS ageing at 90 °C, significant delamination was observed, especially in the thicker films, leading to reduced adhesion forces (19 to 42 N). Control samples held at ambient temperature showed no degradation, confirming that elevated temperature drives failure more than chemical immersion alone. Thermal-shock cycling (−80 °C to +220 °C) also caused interfacial damage: Batch 4 coatings (20 µm) required only ~5.5 N to detach, consistent with microscopy showing near-complete delamination after cycling.

Taken together, these results highlight two critical design considerations for implant coatings: (1) the adhesion promoter and parylene C must withstand prolonged thermal stress above its continuous-use limit (~80 °C), and (2) thicker coatings do not necessarily confer greater durability if thermal, corrosive or mechanical defects are already present. Future work should focus on optimizing deposition conditions, exploring alternate silane coupling agents for enhanced high-temperature adhesion, and evaluating multilayer or hybrid barrier architectures to improve the long-term stability of parylene C/Titanium systems in demanding biomedical environments. In parallel, it would be beneficial to expand the scope of study to include alternative materials and polymers, which offer significantly greater thermal resistance. This could open pathways for protective coatings better suited to withstand extreme thermal cycling or sterilization protocols, such as those used in thermal bonding or sealing processes, without compromising adhesion or mechanical performance while remaining compatible with implantable medical devices and resilient in bacterial and biologically active environments.

## Figures and Tables

**Table 1:** Reflectometry measurements.

Reflectometry Measurements		
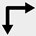	Mean Thickness (µm)	Standard Deviation (µm)
**Batch 1**	**0.87**	**0.17**
**Batch 2**	**4.55**	**0.05**
**Batch 3**	**9.38**	**0.11**
**Batch 4**	**19.17**	**0.13**

**Table 2:** Thickness measurement results using ellipsometry.

Ellipsometry Measurements		
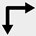	Mean Thickness (µm)	Standard Deviation (µm)
**Batch 1**	**0.78**	**0.04**
**Batch 2**	**4.46**	**0.58**
**Batch 3**	**9.03**	**0.06**
**Batch 4**	**NA**	**NA**

**Table 3:** Interferometry measurements.

Interferometry Measurements		
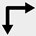	Mean Thickness (µm)	Standard Deviation (µm)
**Batch 1**	**0.85**	**0.03**
**Batch 2**	**4.7**	**0.1**
**Batch 3**	**9.44**	**0.03**
**Batch 4**	**19.26**	**0.45**

**Table 4:** Pull test results.

Interferometry Measurements		
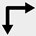	Mean Thickness (µm)	Standard Deviation (µm)
**Batch 1**	**0.85**	**0.03**
**Batch 2**	**4.7**	**0.1**
**Batch 3**	**9.44**	**0.03**
**Batch 4**	**19.26**	**0.45**

**Table 5:** Post corrosion pull test results.

Pull Test Results			
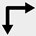	Traction Force (N)	Standard Deviation (N)	Stress (MPa)
**Batch 1**	**31.68**	**9.42**	**1.61**
**Batch 2**	**41.55**	**15.07**	**2.12**
**Batch 3**	**23.08**	**7.14**	**1.18**
**Batch 4**	**19.79**	**8.25**	**1.01**

**Table 6:** Post thermal shock pull test results.

Pull Test Results			
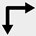	Traction Force (N)	Standard Deviation (N)	Stress (MPa)
**Batch 1**	**31.68**	**9.42**	**1.61**
**Batch 2**	**41.55**	**15.07**	**2.12**
**Batch 3**	**23.08**	**7.14**	**1.18**
**Batch 4**	**19.79**	**8.25**	**1.01**

## Data Availability

The data supporting the findings of this study are available from the corresponding author upon reasonable request.
